# A Novel HCC Prognosis Predictor EEF1E1 Is Related to Immune Infiltration and May Be Involved in EEF1E1/ATM/p53 Signaling

**DOI:** 10.3389/fonc.2021.700972

**Published:** 2021-07-02

**Authors:** Ruiqin Han, Penghui Feng, Junyi Pang, Dingfeng Zou, Xiaolu Li, Chao Geng, Lili Li, Jie Min, Jing Shi

**Affiliations:** ^1^ State Key Laboratory of Medical Molecular Biology, Department of Biochemistry and Molecular Biology, Institute of Basic Medical Sciences, Chinese Academy of Medical Sciences and Peking Union Medical College, Beijing, China; ^2^ Department of Obstetrics and Gynecology, Peking Union Medical College Hospital, Chinese Academy of Medical Sciences and Peking Union Medical College, Beijing, China; ^3^ Department of Pathology, Peking Union Medical College Hospital, Chinese Academy of Medical Sciences and Peking Union Medical College, Beijing, China; ^4^ Beijing Municipal Key Laboratory of Child Development and Nutriomics, Capital Institute of Pediatrics, Beijing, China

**Keywords:** EEF1E1, prognosis, immune infiltration, ATM, P53, HCC

## Abstract

**Background:**

EEF1E1 has been reported to play a role in ovarian cancer, breast cancer, non-small cell lung cancer and other cancers, but its role and mechanism in hepatocellular carcinoma (HCC) are still unknown.

**Methods:**

EEF1E1 expression in human HCC was analyzed *via* the GTEx and TCGA database. Logistic regression analysis was used to analyze the clinicopathological correlation of EEF1E1 expression. The correlation between EEF1E1 expression and patients’ prognosis was analyzed in HCC, shown by forest plots, nomogram and Kaplan–Meier curves. Hazard ratio (HR) with 95% confidence intervals and log-rank *p*-value were calculated *via* multivariate/univariate survival analyses. Moreover, the correlation between EEF1E1 and tumor immune infiltration was analyzed using the gsva package with the ssgsea algorithm. Pearson correlation was used to investigate the correlation between EEF1E1 expression and p53 pathway genes expression. Two third-party databases were used to validate the content of EEF1E1 protein and mRNA expression patterns and prognosis analysis. The immunohistochemistry and multiplex immunohistochemistry was used to verify the bio-informatics results.

**Results:**

EEF1E1 mRNA and protein expression in tumor was statistically higher than normal (EEF1E1 mRNA: *p* < 0.001; EEF1E1 protein: *p* < 0.01). Results from paired t-test (cancer and adjacent tissues) exhibited consistent trend (t = 7.572, *p* < 0.001). Immunohistochemistry showed that EEF1E1 is highly expressed in cancer. The expression of EEF1E1 was positively correlated with body weight, BMI, tumor status, vascular invasion, AFP, logistic grade, T stage and pathological stage. The univariate Cox model revealed that high EEF1E1 expression was strongly associated with worse OS (HR=2.581; 95% CI: 1.782-3.739; *p* < 0.001), as was T stage, pathologic stage, Histologic grade. High EEF1E1 expression was the only independent prognostic factor associated with OS (HR=2.57; 95% CI: 1.715-3.851; *p* < 0.001) in the multivariate analysis. EEF1E1 was significantly correlated with various immune cells, including cytotoxic cells, DC, macrophages, neutrophils, NK cd56bright, TFH, Tgd, Th17, Th2, Treg. Multiplex immunohistochemistry showed that the EEF1E1 protein level is positively correlated to the CD3, CD4, PD1 and is negatively correlated to the CD8. The expression level of EEF1E1 in HCC was significantly correlated with the key genes involved in the p53 pathway. The expression of EEF1E1, ATM, p53 and CASPASE3 in HCC tissues was significantly higher than that in adjacent tissues.

**Conclusion:**

EEF1E1 is highly expressed in cancer tissues in HCC. EEF1E1’s high expression is significantly correlated with worse prognosis and immune cell infiltration of HCC. EEF1E1 may be participating in EEF1E1/ATM/p53 signaling pathway in HCC.

## Introduction

Liver cancer remains a global health challenge, and its incidence rate is increasing year by year (1), which is the second leading cause of cancer-related death worldwide (2). It is estimated that the incidence rate of liver cancer will reach more than one million per year by 2025 (3). Hepatocellular carcinoma (HCC) is the most common type of liver cancer, accounting for about 90% of all liver cancer (4). Because of the high prevalence of hepatitis B virus (HBV) infection, the incidence rate of HCC in Asia and sub-Saharan Africa is the highest (5). Unlike other cancers, the major risk factors associated with HCC are well defined, including viral hepatitis (B and/or C), alcoholism and nonalcoholic fatty liver disease (6). Early diagnosis and treatment are of great significance for the treatment and prognosis of hepatocellular carcinoma. Therefore, it is imperative to screen and identify practical diagnostic and prognostic markers.

Tumor immunity research is one of the frontier hotspots in cancer research (7–9). Tumor cells and related healthy cells form ecosystems that determine disease progression and therapeutic response. The analysis regarding the relationship between tumor and immune cells revealed the ecosystem characteristics associated with immunosuppression and poor prognosis. High frequency of PD-L1 (+) tumor associated macrophages and depleted T cells were found in high-grade ER (+) and ER (-) tumors (10). Traditional dendritic cell type 1 (cdc1) plays an essential role in anti-tumor immunity. Its abundance in the tumor is related to immune-mediated rejection and the success of immunotherapy. The accumulation of cdc1 in mouse tumors is usually dependent on natural killer (NK) cells that produce cdc1, CCL5 and Xcl1 (11). Immunosuppressive tumor microenvironment (TME) is a major obstacle to immunotherapy. In solid tumors, the underlying mechanism for monocytes to preferentially differentiate into immunosuppressive tumor associated macrophages (TAMs) rather than immune-stimulatory dendritic cells (DCS) is still unclear. Using a variety of mouse sarcoma models, it was found that TME can induce tumor cells to produce retinoic acid (RA) and induce monocytes to differentiate into TAMs and away from DC by inhibiting the DC promoting transcription factor IRF4 (11). PD-1 blockade has changed the treatment of advanced clear cell renal cell carcinoma, but the driving factors and resistance of PD-1 response have not been fully elucidated. Compared with non-invasive tumors, invasive tumors lack favorable pbrm1 mutation, and chromosome deletion of 9p21.3 is more unfavorable, which indicates how the potential interaction between immune-phenotype and somatic changes affects the therapeutic effect (12). In a word, the alteration and regulation of the tumor immune microenvironment play a vital role in the occurrence and development of tumor. Revealing the regulatory mechanism of tumor immune microenvironment has a crucial guiding significance for the diagnosis, treatment and prognosis of tumor.

EEF1E1 (Eukaryotic Translation Elongation Factor 1 Epsilon 1, also called p18) is a protein-coding gene. It is reported that the EEF1E1 protein is localized in the cytoplasm and nucleus. In the cytoplasm, the encoded protein is an auxiliary component of the macromolecular tRNA synthase complex. EEF1E1 interacts with ATM and ATR. The interaction with ATM, which takes place independently of Tp53, is induced by DNA damage during genotoxic stress or cell growth. The interaction with ATR is enhanced by UV irradiation. However, its mouse homolog has been shown can translocate to the nucleus during DNA damage and play an active role in ATM/ATR mediated p53 activation (13). However, its expression, function and underlying mechanism in HCC is still unclear.

In this study, we used TCGA and UCSC databases to analyze the differential expression of *EEF1E1* in hepatocellular carcinoma (HCC) tissues and its correlation with clinicopathological features. Logistic regression analysis and Cox univariate/multivariate analysis were used to evaluate the possibility of *EEF1E1* as an independent prognostic indicator of HCC. At the same time, the correlation between its expression and tumor immune microenvironment was analyzed. In addition, the correlation between its expression and p53 signal was investigated. Finally, the differential expression of EEF1E1 in HCC and paracancerous tissues was verified by immunohistochemistry. The correlation and subcellular co-localization between EEF1E1 expression and the expression of ATM, p53 and Cas3 in p53 signaling pathway was further confirmed. The immunocytes infiltration was validated by multiplex IHC. Our results show that EEF1E1 is differentially expressed in HCC and paracancerous tissues and is highly expressed in HCC tissues. EEF1E1 can be used as an independent prognostic marker of HCC, and is related to the immune invasion of HCC, and may participate in the occurrence and progression of HCC through EEF1E1/ATM/p53 signal.

## Materials and Methods

### Sample Information and EEF1E1 Expression Analysis in HCC

The clinical characteristics of 374 hepatocellular carcinomas (HCC) patients in the TCGA (https://portal.gdc.cancer.gov/) were downloaded including T stage, N stage, M stage, pathologic stage, tumor status, gender, race, age, weight, BMI, histologic grade, residual tumor, adjacent hepatic tissue inflammation, AFP (ng/ml), Albumin (g/dl) and vascular invasion. RNAseq data in the TPM format from UCSC XENA database (https://xenabrowser.net/datapages/) are processed by the Toil process. The corresponding normal tissue data of HCC (hepatocellular carcinoma) and GTEX of TCGA were extracted, consisted of normal (160) and tumor (371) samples. RNAseq data in TPM (transcripts per million reads) format was transformed into log2, and then expression comparison among samples was performed. TCGA (https://portal.gdc.cancer.gov/) RNAseq data in Level 3 HTseq FPKM format in HCC project were filtered, and the paired samples were retained. RNAseq data in FPKM (fragments per kilobase per million) format was converted into TPM (transcripts per million reads) format, and log2 transformation was performed to compare the expression among samples. The immunohistochemistry of EEF1E1 expression in HCC was downloaded from Human Protein Atlas (14) (http://www.proteinatlas.org).

### Prognosis Analysis

The correlation between EEF1E1 expression and patients’ prognosis, such as overall survival (OS), disease-free interval (DFI), and progression-free interval (PFI), was analyzed in HCC and shown by forest plots, nomogram and Kaplan–Meier curves. Hazard ratio (HR) with 95% confidence intervals and log-rank p-value were calculated *via* univariate survival analysis.

### Third-Party Database Validation of EEF1E1 Protein and mRNA Expression Patterns and Prognosis Analysis

To confirm the expression patterns and clinical significance of EEF1E1, gene expression profiles of HCC samples (including 202 normal controls and 243 tumor specimens) were retrieved and obtained from the project of the International Cancer Genome Consortium (ICGC) database (https://dcc.icgc.org/) (15). Corresponding clinical information was processed for Kaplan-Meier analysis by log-rank test using the survival package.

Besides, to identify the difference of the protein expression EEF1E1 between two groups, 330 cases of HCC and para-carcinoma tissues were analyzed based on normalized data from the Clinical proteomic Tumor Analysis Consortium (CPTAC) (https://cptac-data-portal.georgetown.edu/datasets).

### Immunohistochemistry

Twenty-two HCC samples were gifted from Junyi Pang of Peking Union Medical College Hospital, and the samples were used in strict accordance with the relevant regulations of the ethics committee of Peking Union Medical College Hospital. Twenty-two samples were fixed with 4% PFA, dehydrated with ascending gradient alcohol, transparent with xylene and embedded in paraffin. Two samples were fixed with 4% PFA, dehydrated with ascending gradient alcohol, transparent with xylene and embedded in paraffin. The paraffin blocks were made into 4 μm thick serial sections and stained by Leica bond RX automatic immunostaining machine, and DAB refine kit according to the standard immunohistochemistry procedure. Zeiss microscope was used for imaging. The primary antibody information and dilution ratio were as follows:

rabbit polyclonal EEF1E1 (NBP1-32184), 1:1000; rabbit polyclonal ATM (ab81292), 1:1000; rabbit polyclonal P53 (ab1101), 1:1000; rabbit polyclonal CASPASE3 (ab184787), 1:5000.

### Validation of Immune Infiltration by Multiplex Immunohistochemistry

CD3, CD4, CD8, and pD1 were simultaneously detected in the same 4 μm thick serial sections and stained by the Leica bond RX automatic immunostaining machine by use of the Opal 7-Color Automation IHC Kit (NEL810001KT), operated according to the standard protocol. And photoed and analyzed by the automatic quantitative pathological imaging system(Vectra polaris, perkin-Elmer, USA). The primary antibody information and dilution ratio were as follows:

rabbit polyclonal CD3 (abmart-T40008), 1:500; rabbit polyclonal CD4 (abmart-T40009), 1:500; rabbit polyclonal CD8 (abmart-T40010), 1:500; rabbit polyclonal pD1 (abmart-T55229), 1:500.

### Statistical Analysis

Wilcoxon signed-rank test was used to analyze *EEF1E1* expression levels in different normal tissues. Differences in *EEF1E1* expression levels in tumor tissues and normal tissues were evaluated by Wilcoxon signed-rank test. Univariate survival analysis was used to analyze the correlation between *EEF1E1* expression and patients’ survival. Kaplan–Meier methods were used to compare survival by different levels of *EEF1E1* expression. Pearson correlation analysis was calculated between *EEF1E1* expression and immune checkpoint marker level and p53 pathway key gene level. Correlations were considered significant and positive when *p* < 0.05 and r > 0.20. *p* < 0.05 was considered significant for all statistical analyses.

## Results

### Clinical Characteristics of Hepatocellular Carcinoma Patients of TCGA

The clinical characteristics of 374 hepatocellular carcinomas (HCC) patients in the TCGA (https://portal.gdc.cancer.gov/) were downloaded including T stage, N stage, M stage, pathologic stage, Tumor status, Gender, Race, Age, Weight, BMI, Histologic grade, Residual tumor, Adjacent hepatic tissue inflammation, AFP (ng/ml), Albumin (g/dl), and Vascular invasion (**Table 1**).

**Table 1:** TCGA liver cancer patient characteristics and *EEF1E1* expression correlated with clinical-pathological characteristics (cox regression).

Characteristic	Low expression of *EEF1E1*	High expression of *EEF1E1*	*p* value
**N**	187	187	
**T stage, n (%)**			**<0.001**
T1	110 (59.8%)	73 (39%)	
T2	38 (20.7%)	57 (30.5%)	
T3	29 (15.8%)	51 (27.3%)	
T4	7 (3.8%)	6 (3.2%)	
**N stage, n (%)**			0.364
N0	130 (99.2%)	124 (97.6%)	
N1	1 (0.8%)	3 (2.4%)	
**M stage, n (%)**			0.623
M0	131 (99.2%)	137 (97.9%)	
M1	1 (0.8%)	3 (2.1%)	
**Pathological stage, n (%)**			**<0.001**
Stage I	104 (59.8%)	69 (39.2%)	
Stage II	38 (21.8%)	49 (27.8%)	
Stage III	30 (17.2%)	55 (31.2%)	
Stage IV	2 (1.1%)	3 (1.7%)	
**Tumor status, n (%)**			**0.005**
Tumor free	116 (64.4%)	86 (49.1%)	
With tumor	64 (35.6%)	89 (50.9%)	
**Gender, n (%)**			0.825
Female	59 (31.6%)	62 (33.2%)	
Male	128 (68.4%)	125 (66.8%)	
**Race, n (%)**			0.571
Asian	75 (41.4%)	85 (47%)	
Black or African American	9 (5%)	8 (4.4%)	
White	97 (53.6%)	88 (48.6%)	
**Age, n (%)**			0.196
<=60	82 (43.9%)	95 (51.1%)	
>60	105 (56.1%)	91 (48.9%)	
**Weight, n (%)**			**0.022**
<=70	83 (46.9%)	101 (59.8%)	
>70	94 (53.1%)	68 (40.2%)	
**BMI, n (%)**			0.111
<=25	82 (48%)	95 (57.2%)	
>25	89 (52%)	71 (42.8%)	
**Histologic grade, n (%)**			**<0.001**
G1	38 (20.7%)	17 (9.2%)	
G2	96 (52.2%)	82 (44.3%)	
G3	47 (25.5%)	77 (41.6%)	
G4	3 (1.6%)	9 (4.9%)	
**Residual tumor, n (%)**			**0.017**
R0	172 (97.7%)	155 (91.7%)	
R1	4 (2.3%)	13 (7.7%)	
R2	0 (0%)	1 (0.6%)	
**Adjacent hepatic tissue inflammation, n (%)**			0.115
None	72 (54.1%)	46 (44.2%)	
Mild	49 (36.8%)	52 (50%)	
Severe	12 (9%)	6 (5.8%)	
**AFP (ng/ml), n (%)**			**<0.001**
<=400	125 (85%)	90 (67.7%)	
>400	22 (15%)	43 (32.3%)	
**Albumin (g/dl), n (%)**			0.830
<3.5	39 (23.8%)	30 (22.1%)	
>=3.5	125 (76.2%)	106 (77.9%)	
**Vascular invasion, n (%)**			**0.005**
No	123 (72.8%)	85 (57%)	
Yes	46 (27.2%)	64 (43%)	
**Age, median (IQR)**	62 (52.5, 68)	60 (51, 69)	0.363
**Weight, median (IQR)**	73 (61, 86)	68 (58, 78)	0.009
**BMI, median (IQR)**	25.22 (22.28, 29.54)	23.88 (21.14, 27.99)	0.036
**AFP (ng/ml), median (IQR)**	9 (4, 50)	28 (6, 1865)	<0.001
**Albumin (g/dl), median (IQR)**	4 (3.5, 4.3)	4.1 (3.5, 4.4)	0.260

The meaning of the bold values provided under p value column of this table is that the expression of EEF1E1 was positively correlated with the T stage, Pathological stage, Tumor status, Weight, Histologic grade, Residual tumor, AFP, and Vascular invasion.

### EEFIE1 mRNA and Protein Is Highly Expressed in the HCC Cancer Tissues

**Figure 1:**
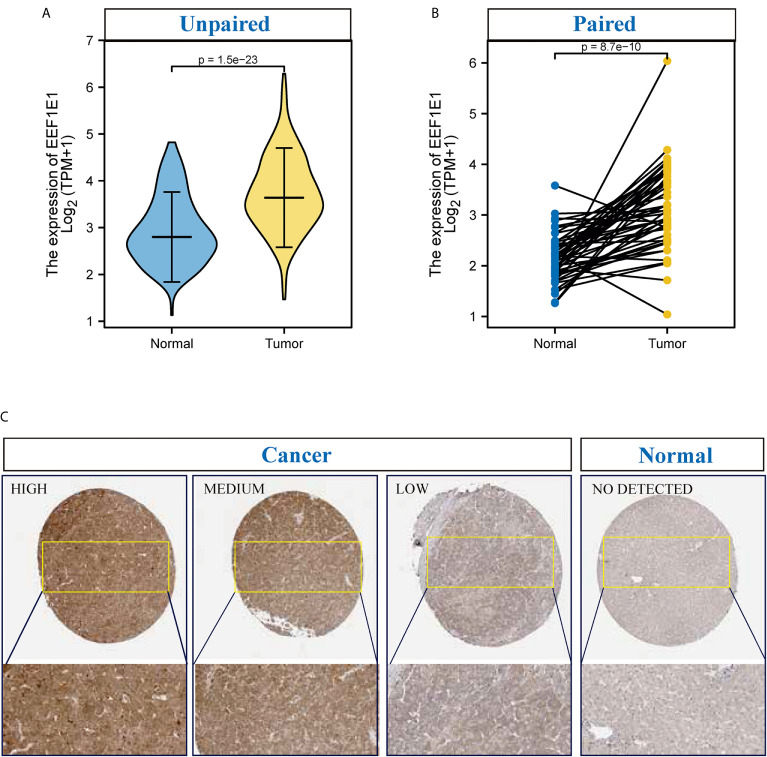
The expression of *EEF1E1* in HCC. **(A)** Comparing the expression of *EEF1E1* in cancer and normal. **(B)** Comparing the expression of *EEF1E1* in cancer and paracancerous. **(C)** The expression of EEF1E1 in HCC from Human protein Atlas.

UCSC XENA (https://xenabrowser.net/datapages/) RNAseq data in TPM format of TCGA and GTEX are processed by the Toil process. The corresponding normal tissue data of HCC (hepatocellular carcinoma) and GTEX of TCGA were extracted, Normal (160) and Tumor (371), respectively. RNAseq data in TPM (transcripts per million reads) format was transformed into log2, and then expression comparison among samples was performed. Mann Whitney U test showed that *EEF1E1* expression in the tumor was higher than normal, the median difference between the two groups was 0.812 (0.671-0.951), the difference was statistically significant (*p* < 0.001) (**Figure 1A**).

TCGA (https://portal.gdc.cancer.gov/) RNAseq data in Level 3 HTseq FPKM format in the HCC project were filtered, and the paired samples were retained. RNAseq data in FPKM (fragments per kilobase per million) format was converted into TPM (transcripts per million reads) format, and log2 transformation was performed to compare the expression among samples. Paired sample (Normal (50) and paracancerous (50)) t-test showed that tumor was higher than the average level of normal, the difference between the two groups was 1.019 (0.748-1.289), the difference was statistically significant (t = 7.572, *p* < 0.001) (**Figure 1B**).

To confirm the expression patterns and clinical significance of *EEF1E1*, gene expression profiles of new hepatocellular carcinoma cohorts (including 202 normal controls and 243 tumor specimens) were retrieved and obtained from the project of the International Cancer Genome Consortium (ICGC) database (https://dcc.icgc.org/). Related clinical information was processed for Kaplan-Meier analysis by log-rank test using the survival package. It was showed that the *EEF1E1* expression level in cancer is significantly higher than that of normal, *p* < 0.01 (**Supplementary Figure 1A**).

It is reported that EEF1E1 is localized in the cytoplasm and nucleus. In the cytoplasm, the encoded protein is an auxiliary component of the macromolecular tRNA synthase complex. However, its mouse homolog has been shown to translocate to the nucleus during DNA damage and play an active role in ATM/ATR mediated p53 activation.

To identify the difference of the EEF1E1 protein expression between two groups (cancer and normal, cancer and paracancerous), 330 cases of hepatocellular carcinoma and paracanerous tissues were analyzed based on normalized data from the Clinical proteomic Tumor Analysis Consortium (CpTAC) (https://cptac-data-portal.georgetown.edu/datasets). The results showed that the expression of EEF1E1 protein in cancer tissues was significantly higher than that in control tissue, *p* < 0.01. Similarly, the expression of EEF1E1 protein in cancer tissues was significantly higher than that in adjacent tissues, *p* < 0.01 (**Figure 1C** and **Supplementary Figures 1C, D**).

To further validate the results for *EEF1E1* expression, 22 cases of HCC sample contains cancer and paracancerous were collected and detected the EEF1E1 expression by IHC. We found that EEF1E1 was expressed in both the nucleus and cytoplasm of HCC cells, but mainly in the cytoplasm, which was consistent with the literature. Meanwhile, the EEF1E1 protein level in cancer is significantly higher than paracancerous (**Supplementary Figures 2**–**6**).

### Relationship Between *EEF1E1* Expression and Clinicopathological Variables in HCC Patients

**Figure 2:**
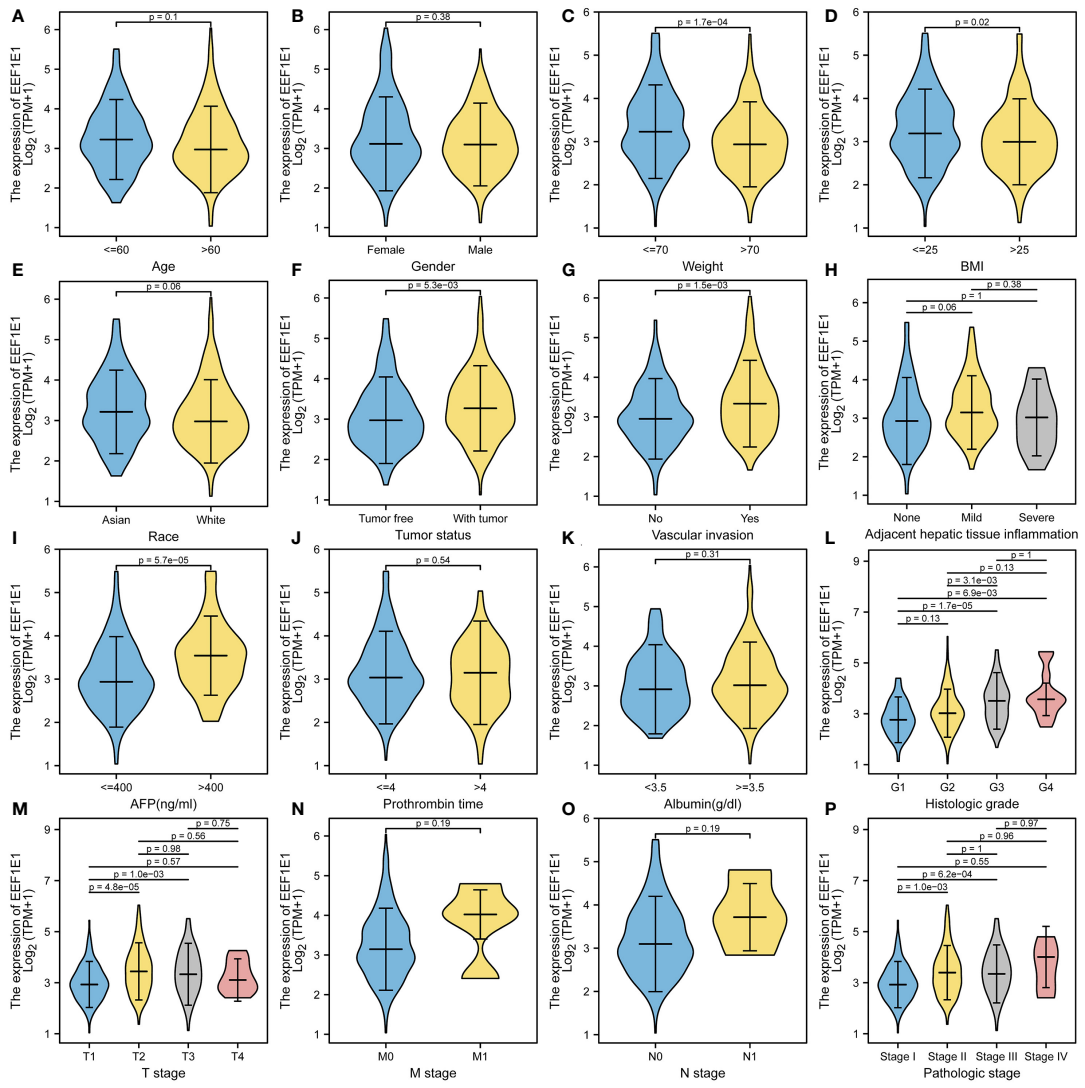
Correlation between expression of EEF1E1 and clinic-pathological features. **(A)** Correlation between expression of EEF1E1 and age. **(B)** Correlation between expression of EEF1E1 and gender. **(C)** Correlation between expression of EEF1E1 and weight. **(D)** Correlation between expression of EEF1E1 and BMI. **(E)** Correlation between expression of EEF1E1 and race. **(F)** Correlation between expression of EEF1E1 and tumor status. **(G)** Correlation between expression of EEF1E1 and vascular invasion. **(H)** Correlation between expression of EEF1E1 and adjacent hepatic tissue inflammation. **(I)** Correlation between expression of EEF1E1 and AFP level. **(J)** Correlation between expression of EEF1E1 and prothrombin time. **(K)** Correlation between expression of EEF1E1 and Albumin level. **(L)** Correlation between expression of EEF1E1 and histologic grade. **(M)** Correlation between expression of EEF1E1 and T stage. **(N)** Correlation between expression of EEF1E1 and M stage. **(O)** Correlation between expression of EEF1E1 and N stage. **(P)** Correlation between expression of EEF1E1 and pathologic stage.

**Figure 3:**
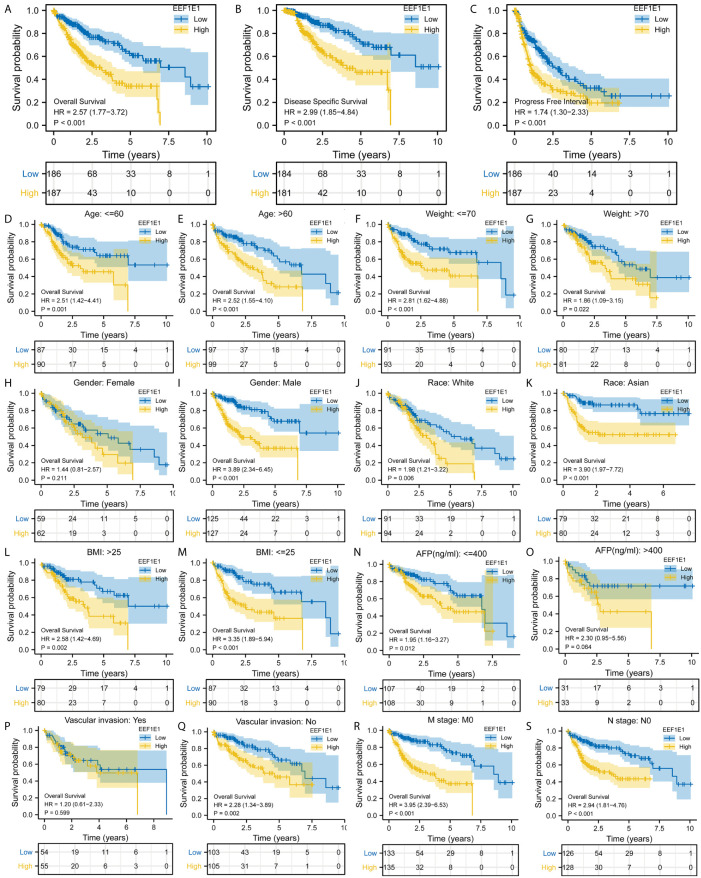
Correlation between EEF1E1 expression and prognosis. **(A)** Effect of differential EEF1E1 expression on overall survival. **(B)** Effect of differential EEF1E1 expression on disease specific survival. **(C)** Effect of differential EEF1E1 expression on overall progress free interval. **(D, E)** Effect of differential EEF1E1 expression and age on overall survival. **(F, G)** Effect of differential EEF1E1 expression and weight on overall survival. **(H, I)** Effect of differential EEF1E1 expression and gender on overall survival. **(J, K)** Effect of differential EEF1E1 expression and race on overall survival. **(L, M)** Effect of differential EEF1E1 expression and BMI on overall survival. **(N, O)** Effect of differential EEF1E1 expression and AFP level on overall survival. **(P, Q)** Effect of differential EEF1E1 expression and vascular invasion on overall survival. **(R)** Effect of differential EEF1E1 expression and M stage on overall survival. **(S)** Effect of differential EEF1E1 expression and N stage on overall survival.

**Figure 4:**
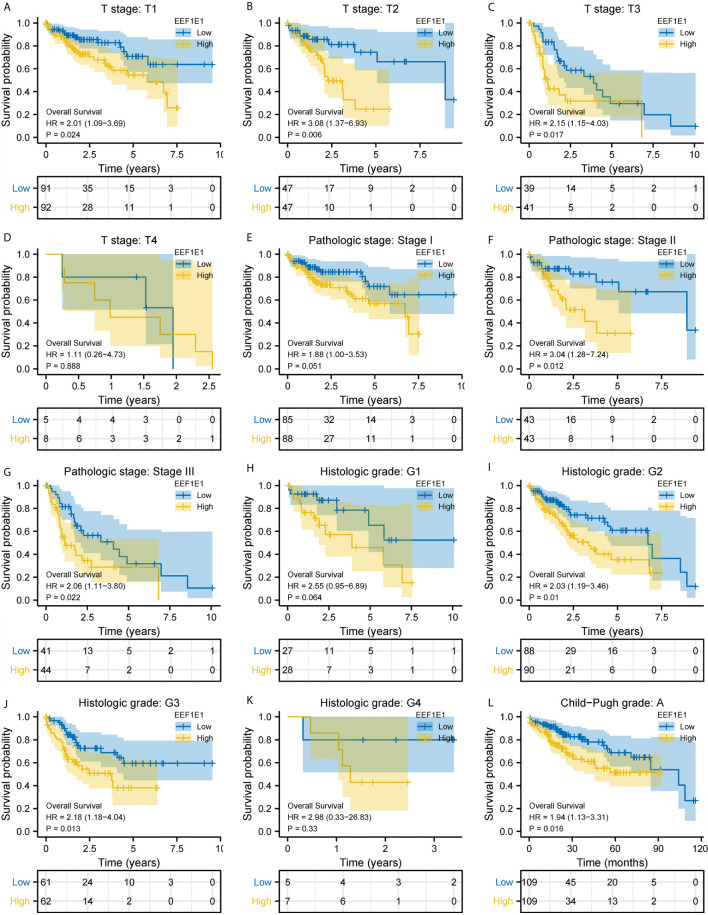
Correlation between EEF1E1 expression and prognosis (continued). **(A–D)** Effect of differential EEF1E1 expression and T stage on overall survival. **(E–G)** Effect of differential EEF1E1 expression and pathologic stage on overall survival. **(H–K)** Effect of differential EEF1E1 expression and histologic grade on overall survival. **(L)** Effect of differential EEF1E1 expression and Child-Pugh grade on overall survival.

**Figure 5:**
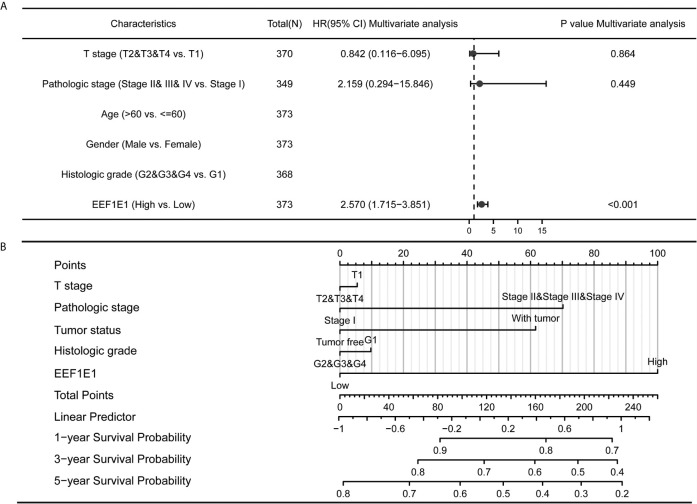
Forest map and nomogram map showed the correlation between EEF1E1 expression and prognosis. **(A)** Forest map showed the correlation between EEF1E1 expression and prognosis. **(B)** Nomogram map showed the correlation between EEF1E1 expression and prognosis.

The expression of *EEF1E1* was positively correlated with the bodyweight of HCC patients. The average level of body weight < 70 group was 3.342 ± 0.84, and the average level of body weight > 70 group was 3.021 ± 0.731. Mann Whitney U test showed that the expression level of *EEF1E1* in patients with body weight > 70 was lower than that in patients with body weight < 70, and the median difference between the two groups was -0.317 (- 0.477-0.14), with statistical significance (*p* < 0.001). The *EEF1E1* expression level was significantly correlated with AFP level, *p* < 0.001. In histological grade, the expression of G1 *EEF1E1* was significantly different from G3 (*p* < 0.001) and G4 (*p* < 0.001), and the expression of G2 *EEF1E1* was significantly different from G3 (*p* = 0.003). In the T stage, the expression of *EEF1E1* in T1 was significantly different from that in T2 (*p* < 0.001) and T3 (*p* = 0.001), but not significantly different from that in other stages. *EEF1E1* expression was not related to M and N stages. The expression of *EEF1E1* in stage I was significantly different from that in stage II (*p* = 0.003) and stage III (*p* < 0.001). The above results showed that the expression level of *EEF1E1* was related to body weight, BMI, tumor status, vascular invasion, AFP, logistic grade, T stage and pathological stage (**Figures 2**–**5** and **Table 2**).

**Table 2:** *EEF1E1* expression correlated with clinical pathological characteristics (logistic regression).

Characteristics	Total (N)	Odds Ratio (OR)	*p* value
T stage (T2&T3&T4 *vs*. T1)	371	2.541 (1.678-3.875)	**<0.001**
N stage (N1 *vs*. N0)	258	3.298 (0.416-67.150)	0.304
M stage (M1 *vs*. M0)	272	2.956 (0.373-60.163)	0.351
Histologic grade (G2&G3&G4 *vs*. G1)	369	2.572 (1.414-4.855)	**0.003**
pathologic stage (Stage II&Stage III&Stage IV *vs*. Stage I)	350	2.599 (1.694-4.016)	**<0.001**
Tumor status (With tumor vs. Tumor free)	355	1.927 (1.261-2.959)	**0.003**
AFP (ng/ml) (>400 *vs*. <=400)	280	2.060 (1.174-3.669)	**0.013**
Vascular invasion (Yes *vs*. No)	318	1.974 (1.238-3.167)	**0.004**

The meaning of the bold values provided under p value column of this table is that the expression of EEF1E1 was positively correlated with the T stage, Pathological stage, Tumor status, Weight, Histologic grade, Residual tumor, AFP, and Vascular invasion by logistic regression.

### EEF1E1 May Be an Independent Prognostic Predictor for HCC

Kaplan Meier survival analysis was used to evaluate the value of EEF1E1 mRNA and protein expression in the prognosis of patients with HCC. The results showed that the expression of *EEF1E1* was correlated with the prognosis of HCC patients. Compared with the patients with low expression of *EEF1E1*, the prognosis of patients with high expression of *EEF1E1* was worse, whether it was overall survival (*p* < 0.001), disease-specific survival (*p* < 0.001), or progress free survival (*p* < 0.001) (**Figure 3**).

Meanwhile, the influence of *EEF1E1* expression level on the prognosis of HCC patients was also related to age (<= 60 & >60, *p* = 0.001), weight (<= 70, *p* < 0.001; > 70, *p* = 0.022), gender (Male, *p* < 0.001), race (White, *p* = 0.006; Asian, *p* < 0.001), BMI (>25, *p* = 0.002; <= 25, *p* < 0.001), AFP (<= 400, *p* = 0.01), vascular invasion (NO, *p* = 0.002), M0 (*p* < 0.001), N0 (*p* < 0.001). In addition, the effect of *EEF1E1* expression level on the prognosis of HCC patients was also related to T stage (T1, *p* = 0.024; T2, *p* = 0.006; T3, *p* = 0.017), pathological stage (Stage II, *p* = 0.012; Stage III, *p* = 0.022), histologic grade (G2, *p* = 0.01; G3, *p* = 0.013) and child pugh grade (A, *p* = 0.016) (**Figure 4**). Accordingly, we assessed the *p*rognostic variables correlated with OS using univariate Cox regression analyses (**Table 3**). The univariate Cox model revealed that high *EEF1E1* expression was strongly associated with worse OS (HR = 2.581; 95% CI: 1.782 - 3.739; *p* < 0.001), as was T stage, pathologic stage, Histologic grade. As shown in **Figure 3B**, high *EEF1E1* expression was the only independent prognostic factor associated with OS (HR = 2.57; 95% CI: 1.715 - 3.851; *p* < 0.001) in the multivariate analysis.

**Table 3:** Univariate analysis and multivariate analysis of liver cancer patient’s overall survival.

Characteristics	Total (N)	Univariate analysis		Multivariate analysis	
		Hazard ratio (95% CI)	*p* value	Hazard ratio (95% CI)	*p* value
T stage (T2&T3&T4 *vs*. T1)	370	2.126 (1.481-3.052)	**<0.001**	0.842 (0.116-6.095)	0.864
pathologic stage (Stage II&Stage III&Stage IV *vs*. Stage I)	349	2.090 (1.429-3.055)	**<0.001**	2.159 (0.294-15.846)	0.449
Age (>60 *vs*. <=60)	373	1.205 (0.850-1.708)	0.295		
Gender (Male *vs*. Female)	373	0.793 (0.557-1.130)	0.200		
Histologic grade (G2&G3&G4 *vs*. G1)	368	1.188 (0.721-1.958)	0.499		
*EEF1E1* (High *vs*. Low)	373	2.581 (1.782-3.739)	**<0.001**	2.570 (1.715-3.851)	**<0.001**

The meaning of the bold values provided under p value column of this table is that the T stage, Pathological stage and the EEF1E1 expression level are bad factors to HCC prognosis by univariate analysis, and the EEF1E1 expression level is the independent factor indicates a worse prognosis outcome by multivariate analysis.

To confirm the clinical significance of *EEF1E1*, gene expression profiles of new hepatocellular carcinoma cohorts (including 202 normal controls and 243 tumor specimens) were retrieved and obtained from the project of the International Cancer Genome Consortium (ICGC) database (https://dcc.icgc.org/). Corresponding clinical information was processed for Kaplan-Meier analysis by log-rank test using the survival package. It was showed that the difference of overall survival probability between high and low *EEFIE1* expression group is significant, *p* < 0.01 (**Supplementary Figure 1B**).

To confirm the clinical significance of EEF1E1, 330 cases of hepatocellular carcinoma and paracanerous tissues were analyzed based on normalized data from the Clinical proteomic Tumor Analysis Consortium (CpTAC) (https://cptac-data-portal.georgetown.edu/datasets). It was shown that the overall survival probability of high EEF1E1 protein was significantly lower than that of the low EEF1E1 protein, *p* = 0.023 (**Supplementary Figure 1E**).

### The Expression Level of *EEF1E1* Is Related to Tumor Immune Microenvironment of HCC

Immune correlation analysis and visualization were conducted by software R (version 3.6.3). R package [gsva package (16)] was used for Immune correlation analysis by immune cell algorithm (ssgsea). Immunocytes (15) analyzed including ADC [activated DC]; B cells; CD8 T cells; cytotoxic cells; DC; eosinophils; IDC [emotion DC]; macrophages; mast cells; neutrophils; NK cd56bright cells; NK cd56dim cells; NK cells; pDC [plasmacytoid DC]; T cells; T helper cells; TCM [T central memory]; TEM [T effector memory]; TFH [T follicular helper]; TGD [T gamma delta]; Th1 cells; Th17 cells; Th2 cells and Treg cells. RNAseq data and clinical data in Level 3 HTseq FPKM format in HCC project retrieve from TCGA (https://portal.gdc.cancer.gov/). RNAseq data were filtering by removal of paracancerous tissue. RNAseq data in FPKM (fragments per kilobase per million) format were analyzed after log2 transformation.

**Figure 6:**
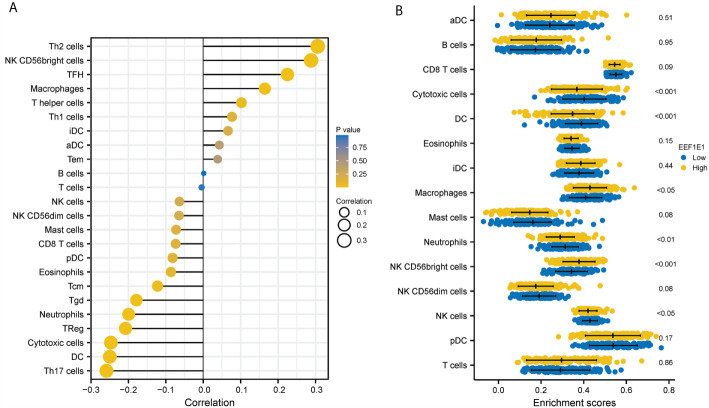
Correlation between expression of EEF1E1 and immune infiltration. **(A)** Correlation between expression of EEF1E1 and immune cells infiltration. **(B)** Effect of differential EEF1E1 expression on immune cells infiltration.

**Figure 7:**
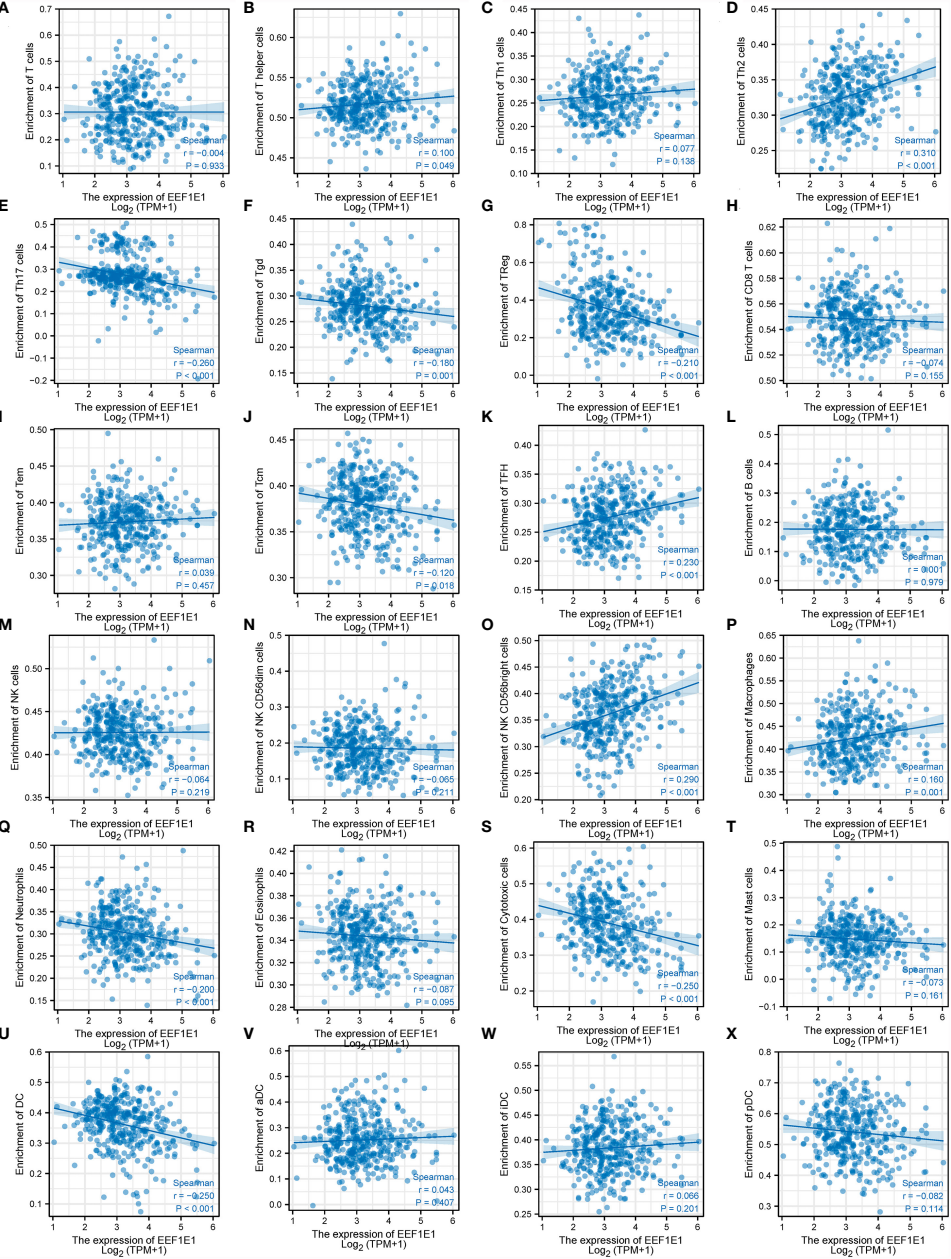
Correlation between expression of EEF1E1 and immune infiltration (continued). **(A–K)** Correlation between expression of EEF1E1 and kinds of T cells infiltration. **(L)** Correlation between expression of EEF1E1 and B cells infiltration. **(M–O)** Correlation between expression of EEF1E1 and kinds of NK cells infiltration. **(P)** Correlation between expression of EEF1E1 and macrophages infiltration. **(Q)** Correlation between expression of EEF1E1 and neutrophils infiltration. **(R)** Correlation between expression of EEF1E1 and eosinophils infiltration. **(S)** Correlation between expression of EEF1E1 and cytotoxic cells infiltration. **(T)** Correlation between expression of EEF1E1 and mast cells infiltration. **(U–X)** Correlation between expression of EEF1E1 and kinds of DCs infiltration.

We used immune cell algorithm (ssgsea) to evaluate the correlation between *EEF1E1* and immune cells, and found that *EEF1E1* was significantly correlated with a variety of immune cells, including cytotoxic cells (r = -0.247, *p* < 0.001), DC (r = -0.250, *p* < 0.001), macrophages (r = 0.165, *p* = 0.001), neutrophils (r = -0.199, *p* < 0.001), NK cd56bright cells (r = 0.288, *p* < 0.001), TFH (r = 0.225, *p* < 0.001), Tgd (r = -0.179, *p* < 0.001), Th17 cells (r = -0.259, *p* < 0.001), Th2 cells (r = 0.306, *p* < 0.001), TReg (r = -0.208, *p* < 0.001). In addition, we compared the enrichment scores of immunocytes with high and low expression of *EEF1E1*. The results showed that the enrichment fraction of cytotoxic cells (*p* < 0.001), DC (*p* < 0.001), neutrophils (*p* < 0.01) and NK cells (*p* < 0.05) with high expression of *EEF1E1* was significantly lower than that with low expression of *EEF1E1*, but the opposite was true in macrophages (*p* < 0.05) and NK CD56bright cells (*p* < 0.001) (**Figures 6**, **7**).

To verify the infiltration of the immune cells analyzed by bio-informatics above, a newly developed multiplex immunohistochemistry technology was used to determine the immune cells infiltrated in 2 cases of hepatocellular carcinoma. The T helper cells marker (CD3, CD4), CD8 T, and PD1 were investigated, it was found that EEF1E1 protein level is positively correlated to the CD3, CD4, PD1 and is negatively correlated to the CD8. This was consistent with the results of bioinformatics (**Supplementary Figures 10**, **11**).

### The Expression Level of EEF1E1 Is Related to EEF1E1/ATM/p53 Signaling Pathway

It is reported that EEF1E1 protein is localized in the cytoplasm and nucleus. In the cytoplasm, the encoded protein is an auxiliary component of the macromolecular tRNA synthase complex. EEF1E1 interacts with ATM and ATR. The interaction with ATM, which takes place independently of Tp53, is induced by DNA damage during genotoxic stress or cell growth. The interaction with ATR is enhanced by UV irradiation. However, its mouse homolog has been shown to translocate to the nucleus during DNA damage and play an active role in ATM/ATR mediated p53 activation (The haploinsufficient tumor suppressor p18 upregulates p53 *via* interactions with ATM/ATR (13).

**Figure 8:**
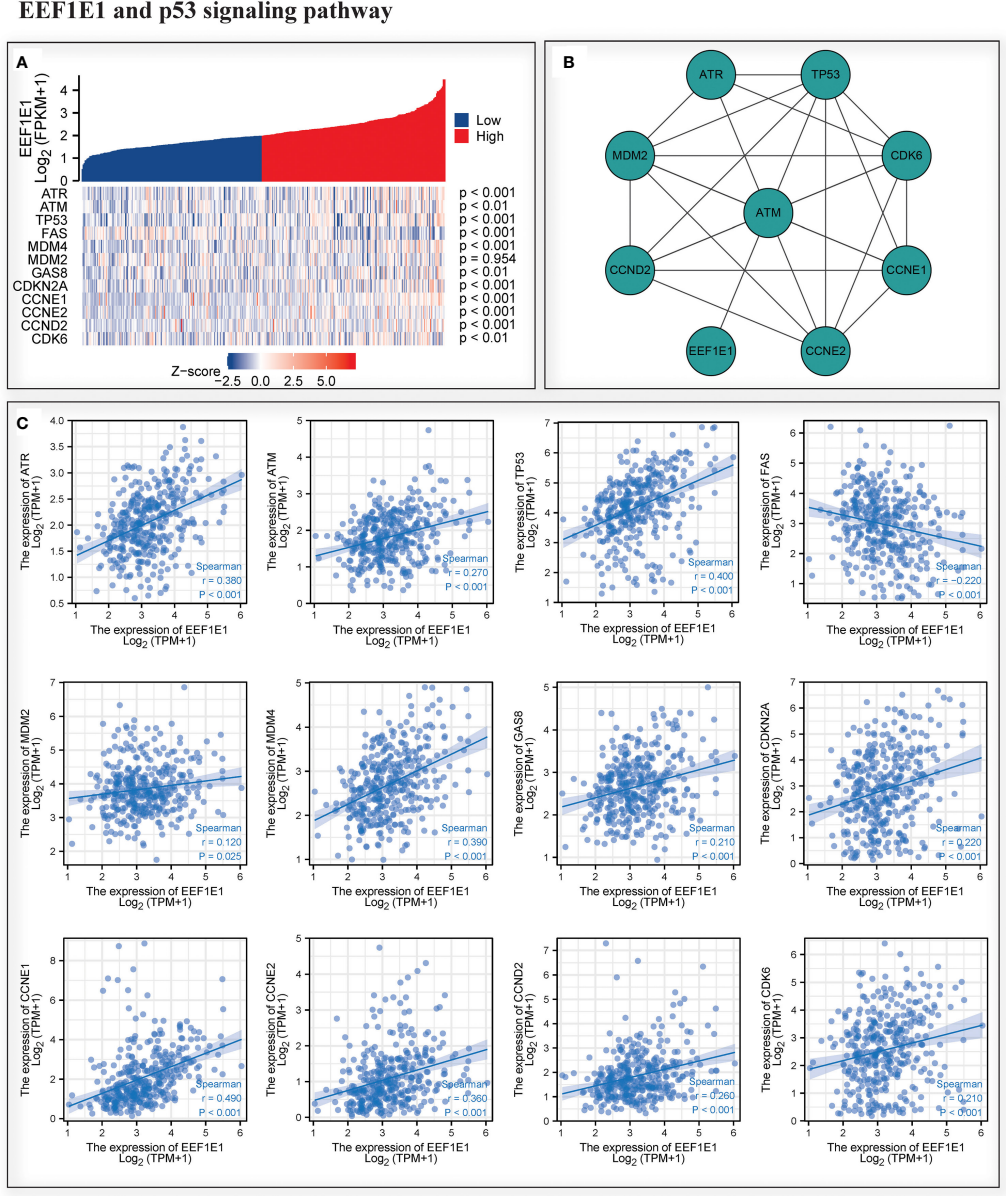
Correlation between the expression of EEF1E1 and p53 pathway. **(A)** Correlation between the expression of EEF1E1 and p53 pathway key molecules expression level. **(B)** Network map showing the interaction of EEF1E1 and p53 pathway key molecules. **(C)** Correlation between the expression of EEF1E1 and p53 pathway key molecules expression level.

We analyzed the correlation between *EEF1E1* expression and *p53* pathway by gene co-expression analysis and gene association analysis. It was found that the expression level of *EEF1E1* in HCC was significantly correlated with *ATR, ATM, Tp53, FAS, MDM4, GAS8, CDKN2A, CCNE1, CCNE2* and *CDK6* (**Figures 8A, C**). The results of PPI and Cytoscape analysis showed that EEF1E1 might be the upstream gene of p53 signal and mediate p53 pathway by regulating ATM (**Figure 8B**).

**Figure 9:**
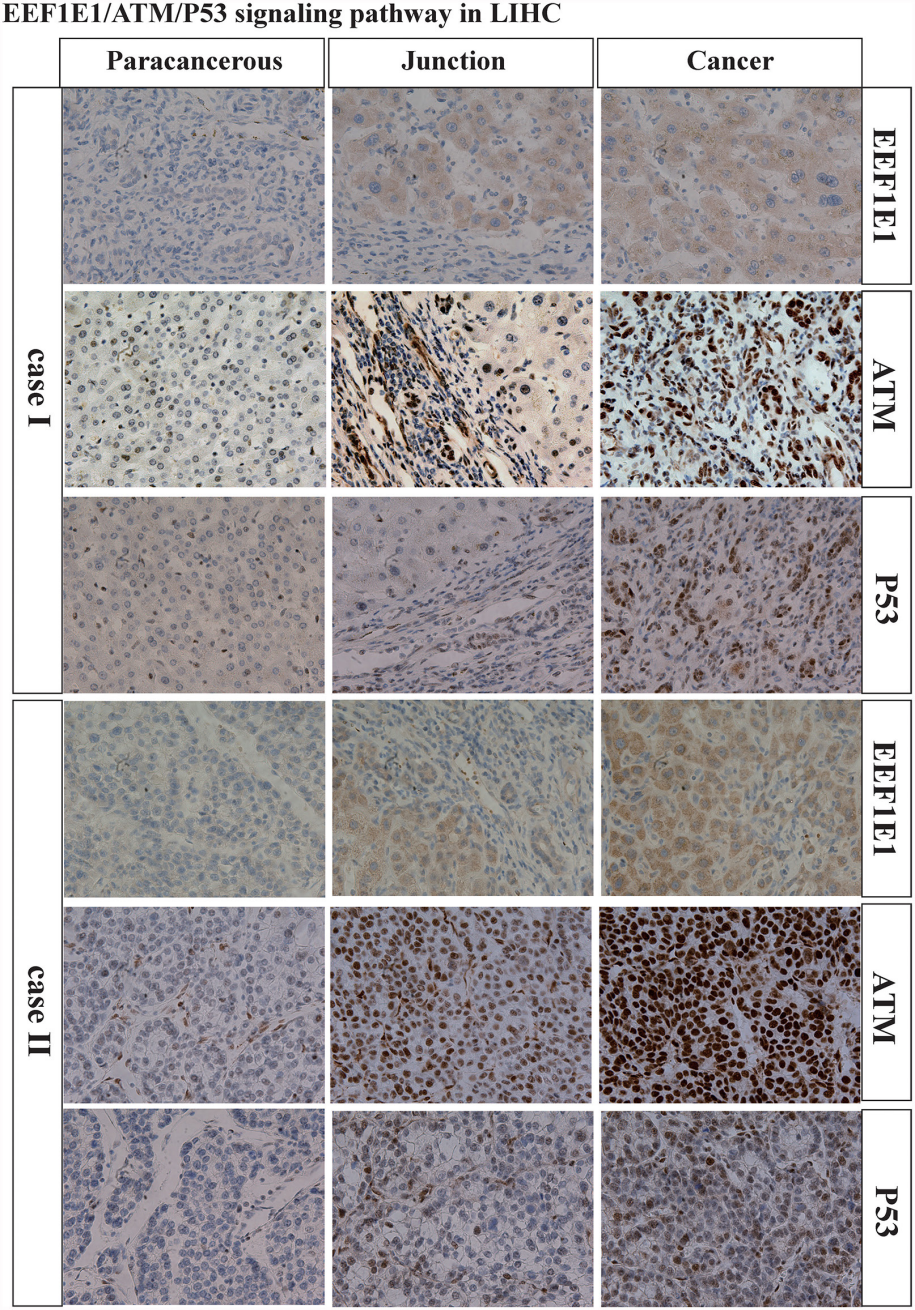
Immunohistochemically verification of bioinformatics analysis.

We examined the expression of EEF1E1, ATM, p53 and CASPASE3 in two cases of HCC by immunohistochemistry. We found that the expression of EEF1E1, ATM, p53 and CASPASE3 in HCC tissues was significantly higher than that in adjacent tissues. In addition, EEF1E1 was expressed in the cytoplasm and nucleus of HCC cells, and mainly expressed in the cytoplasm, while ATM and p53 were represented in the nucleus of HCC cells (**Supplementary Figures 7**–**9** and **Figure 9**).

## Discussion

EEF1E1 has been reported to play a role in ovarian cancer (17), breast cancer (18, 19), non-small cell lung cancer (19) and other cancers (20–22), but its role and mechanism in hepatocellular carcinoma is still unclear. Our results show that EEF1E1 mRNA and protein is deferentially expressed in HCC and paracancerous tissues and is highly expressed in HCC tissues. EEF1E1 can be used as an independent prognostic marker of HCC, and is related to the immune invasion of HCC, and may participate in the occurrence and progression of HCC through EEF1E1/ATM/p53 signal. In epithelial ovarian cancer, YB-1/p18 levels were significantly decreased in older patients (*p* = 0.021). In our study, there is no significant difference related to age. Studies on the anticancer mechanism of peptide p18 in human leukemia K562 cells revealed that p18 causes the death of most K562 cells by depolarizing plasma membrane potential and enhancing membrane permeability, rather than activating the classical apoptosis pathway (23). p18 (INK4c), a member of the INK4 family of cyclin-dependent kinase inhibitors, negatively regulates the cyclin D-cyclin-dependent kinase 4/6 complexes which promote G1/S transition by phosphorylating the retinoblastoma tumor-suppressor gene product. Several recent studies using p18 (INK4c)-null mice revealed that p18 (INK4c) plays a vital role in cell proliferation and tumor development. It was reported that 12-O-tetradecanoylphorbol-13-acetate (TpA), widely used as a protein kinase C (pKC) activator, suppresses the expression of p18 (INK4c) through its promoter, accompanied by the induction of human cancer cell growth. Reduction of p18 (INK4c) using small interfering RNA (siRNA) also enhanced cell growth, suggesting that p18 (INK4c) is a critical target of TpA. Ro31-8425, a potent and highly specific pKC inhibitor abrogated the suppressive effect of TpA on p18 (INK4c) gene expression. However, the expression of dominant-negative c-Jun (TAM-67) did not inhibit the action of TpA on p18 (INK4c). These findings suggest that activation of pKC promotes human cancer cell growth through downregulation of p18 (INK4c) in an Ap-1 activation-independent manner. These results indicate that the accelerated cellular proliferation of some human tumors caused by enhanced pKC activity at least partially involves the suppression of p18 (INK4c), which is a ubiquitously expressed cyclin-dependent kinase inhibitor (24). The p15 (INK4B), p16 (INK4) and p18 genes are members of the gene family coding for inhibitors of cyclin-dependent kinases 4 and 6. To examine the role of these three genes in lung carcinogenesis, somatic mutations within the genes were analyzed by single-strand conformation polymorphism and DNA sequencing in 71 non-small-cell lung cancer (NSCLC) samples. Six somatic mutations in the p16 (INK4) gene and 3 cases with a polymorphic allele were observed. Loss of heterozygosity in the p18 gene was found in 1 sample, did not find any intragenic mutations in the p15 (INK4B) or p18 genes. p16 (INK4) mutations play a role in the formation of some NSCLCs, whereas the involvement of p15 (INK4B) and p18 is uncommon (19). Cyclin-dependent kinase-4 inhibitor genes (INK4) regulate the cell cycle and are candidate tumor-suppressor genes. To determine if alterations in the coding regions of the p18 and p19 genes, which are novel members of the INK4 family and if they correlate with the development of human cancer, 100 human cancer cell lines were analyzed. Neither homozygous deletions nor intragenic mutations of the p18 and p19 genes were found except in an ovarian cancer cell line, SKOV3, harboring a single base pair deletion in exon 1 of p19. Using Western blot analysis, subsets of 26 human cancer cell lines were examined for p18 expression and 39 cell lines for p19 expression. All of these cell lines expressed the p18 or p19 protein, except for SKOV3, which did not express p19. The group includes p18 and p19, in which somatic mutations are uncommon in many types of human cancer, and their role in human carcinogenesis and cancer progression is uncertain (25).

In summary, the present study has indicated that *EEF1E1* overexpression correlates with poor prognosis and increases immune infiltration levels in cytotoxic cells (r = -0.247, *p* < 0.001), DC (r = -0.250, *p* < 0.001), macrophages (r = 0.165, *p* = 0.001), neutrophils (r = -0.199, *p* < 0.001), NK cd56bright cells (r=0.288, *p* < 0.001), TFH (r=0.225, *p* < 0.001), Tgd (r=-0.179, *p* < 0.001), Th17 cells (r=-0.259, *p* < 0.001), Th2 cells (r=0.306, *p* < 0.001) and TReg (r=-0.208, *p <*0.001) in HCC. In addition, in HCC, *EEF1E1* expression was strongly correlated with the p53 signaling. However, these results were based on data analysis. Immunohistochemistry and multipex immunohistochemistry was used for preliminary verification. Further experimental verification will be carried out in our next study. Therefore, EEF1E1 may play an important role in tumor immunity and be a potential prognosis biomarker in patients with HCC and may be involved in EEF1E1/ATM/p53 signaling pathway.

## Data Availability Statement

Publicly available datasets were analyzed in this study. This data can be found here: TCGA (https://portal.gdc.cancer.gov/) UCSC XENA (https://xenabrowser.net/datapages/).

## Ethics Statement

The studies involving human participants were reviewed and approved by the ethics committee of Peking Union Medical College Hospital. Written informed consent for participation was not required for this study in accordance with the national legislation and the institutional requirements.

## Author Contributions

RQH designed and supervised the study. RQH analyzed the data and wrote the original draft. PHF conducted the third-party database validation. JYP provided the HCC samples. JYP, DFZ, and JS edited the draft. XLL, CG, LLL, and JM reviewed the draft. All authors contributed to the article and approved the submitted version.

## Funding

This study was supported by the National Natural Science Foundation of China (grant No. 81801433) and State Key Laboratory Special Fund of China (grant No. 2060204).

## Conflict of Interest

The authors declare that the research was conducted in the absence of any commercial or financial relationships that could be construed as a potential conflict of interest.

## Supplementary Material

Supplementary Figure 1 Validation of the expression patterns and prognosis significant of EEF1E mRNA and protein by use of third-party database.

Supplementary Figure 2 The expression of EEF1E1 in HCC was detected by IHC.

Supplementary Figure 3 The expression of EEF1E1 in HCC was detected by IHC(continued).

Supplementary Figure 4 The expression of EEF1E1 in HCC was detected by IHC(continued).

Supplementary Figure 5 The expression of EEF1E1 in HCC was detected by IHC(continued).

Supplementary Figure 6 The expression of EEF1E1 in HCC was detected by IHC(continued).

Supplementary Figure 7 The expression of ATM in HCC was detected by IHC.

Supplementary Figure 8 The expression of p53 in HCC was detected by IHC.

Supplementary Figure 9 The expression of CASPASE3 in HCC was detected by IHC.

Supplementary Figure 10 Multiplex IHC detection the expression of CD3, CD4, CD8 and PD1 in HCC.

Supplementary Figure 11 Multiplex IHC detection the expression of CD3, CD4, CD8 and PD1 in HCC(continued).
