# Supplementary figures and images for: A Novel HCC Prognosis Predictor EEF1E1 Is Related to Immune Infiltration and May Be Involved in EEF1E1/ATM/p53 Signaling
Source: Front Oncol. 2021 Jul 2;11:700972. doi: 10.3389/fonc.2021.700972 (PMC8285289; doi:10.3389/fonc.2021.700972)

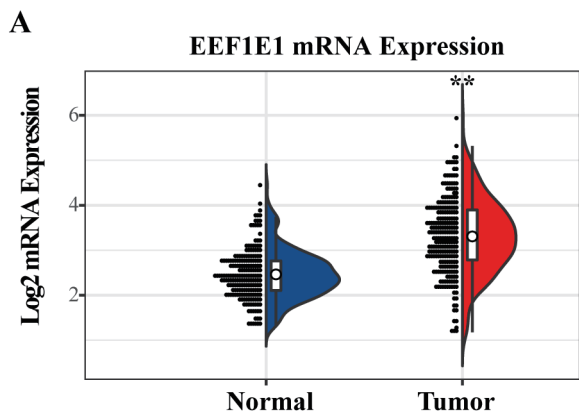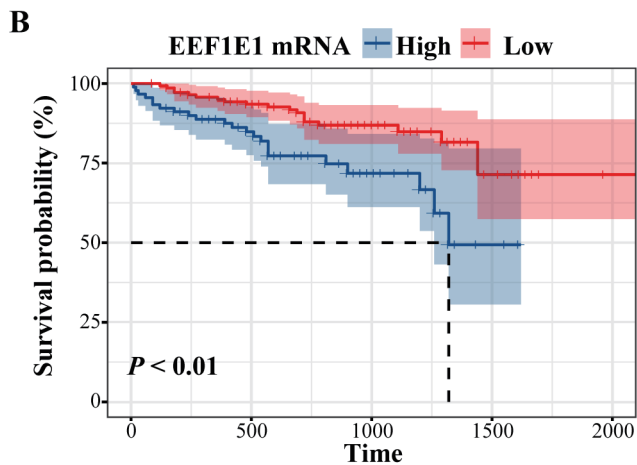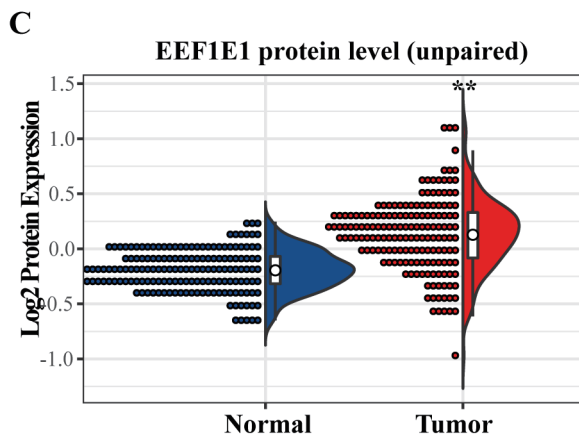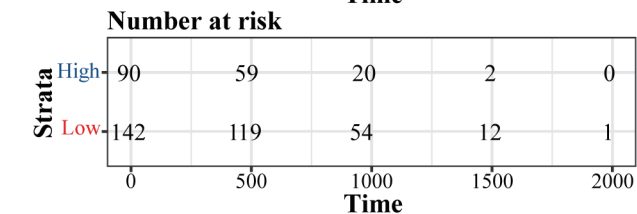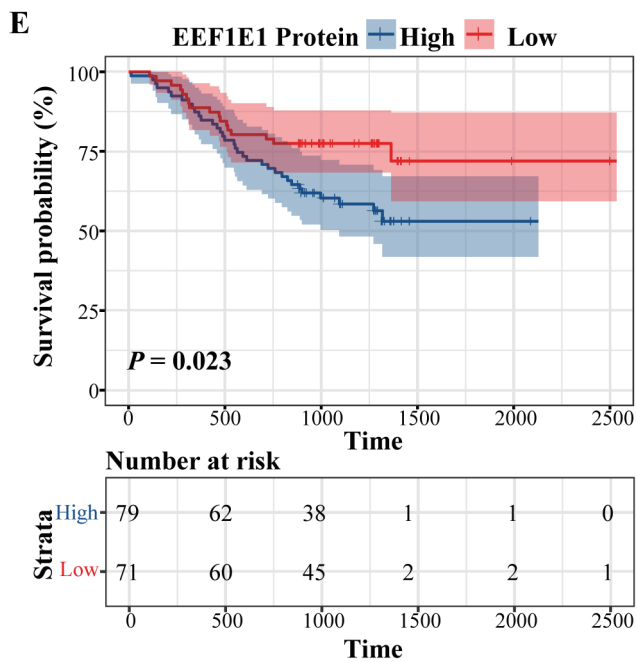

Supplement: Supplementary Figure 1 — Validation of the expression patterns and prognosis significant of EEF1E mRNA and protein by use of third-party database. [file DataSheet_1.pdf]

EEF1E1 expression in LIHC tissues

|         | Paracancerous                                                                       | Junction                                                                            | Cancer                                                                               |      |
|---------|-------------------------------------------------------------------------------------|-------------------------------------------------------------------------------------|--------------------------------------------------------------------------------------|------|
| case I  | 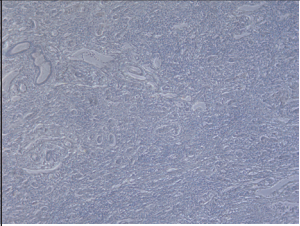   | 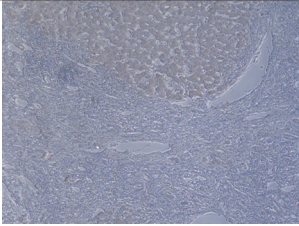   | 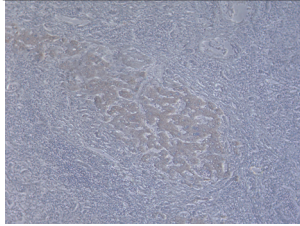   | 10 x |
|         | 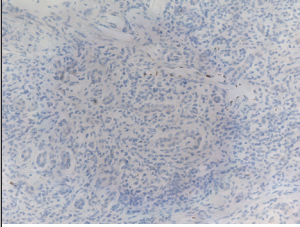   | 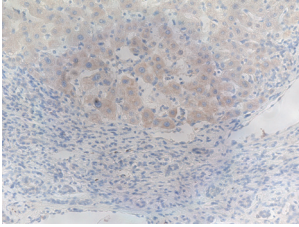   | 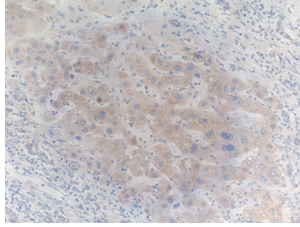   | 20 x |
|         | 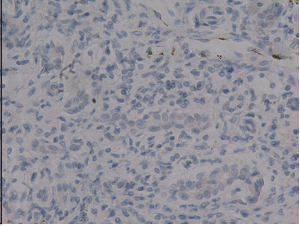   | 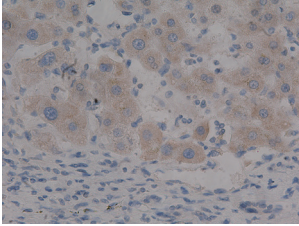   | 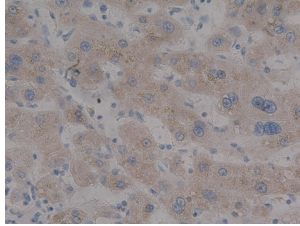   | 40 x |
| case II | 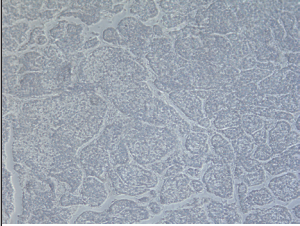  | 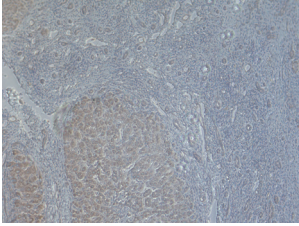  | 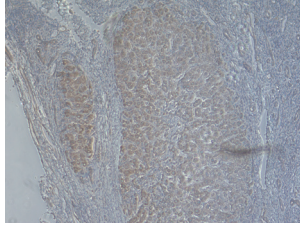  | 10 x |
|         | 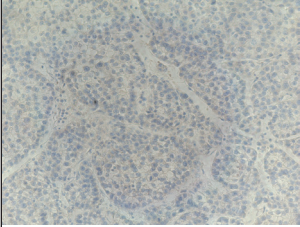 | 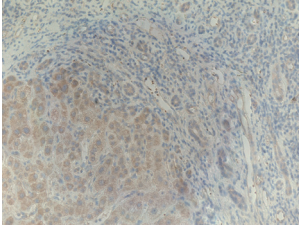 | 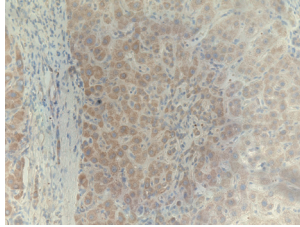 | 20 x |
|         | 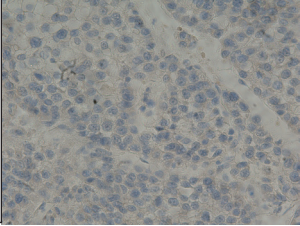 | 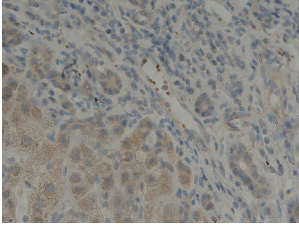 | 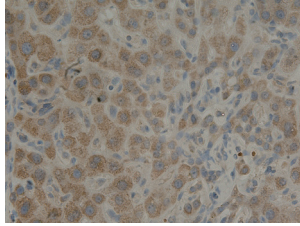 | 40 x |

Supplement: Supplementary Figure 2 — The expression of EEF1E1 in HCC was detected by IHC. [file DataSheet_2.pdf]

|          | Cancer                                                                              | Paracancerous                                                                        |
|----------|-------------------------------------------------------------------------------------|--------------------------------------------------------------------------------------|
| Case I   | 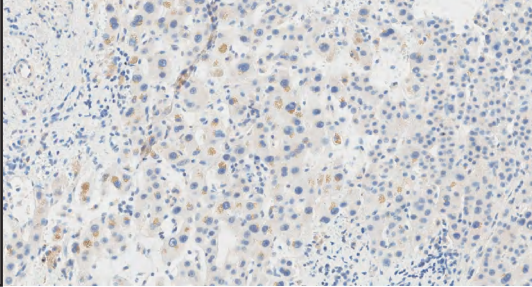    | 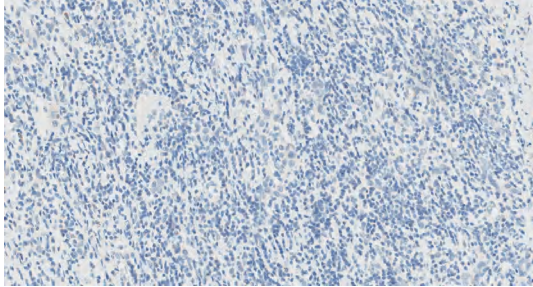    |
| Case II  | 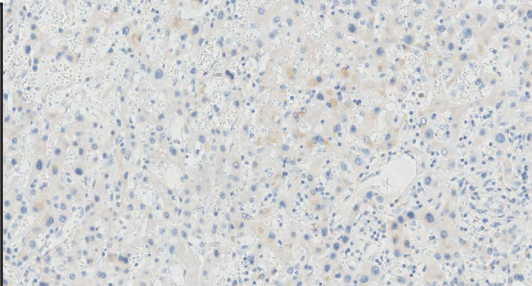   | 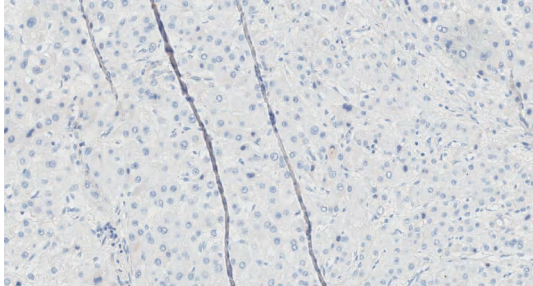   |
| Case III | 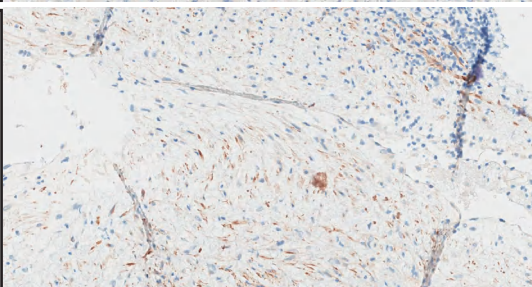   | 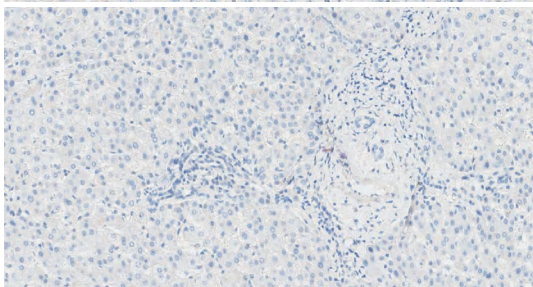   |
| Case IV  | 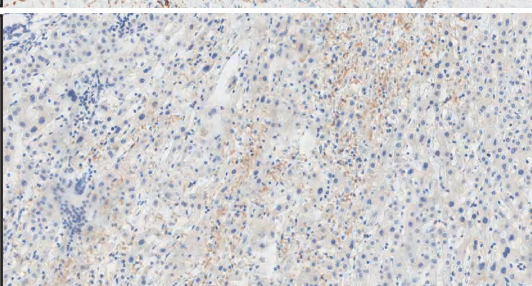  | 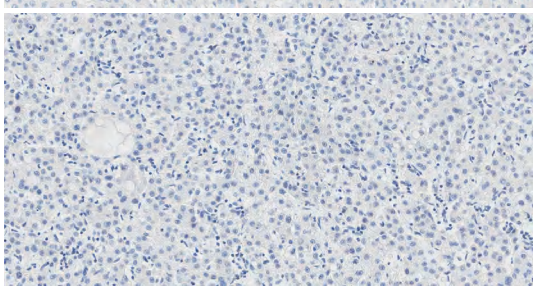  |
| Case V   | 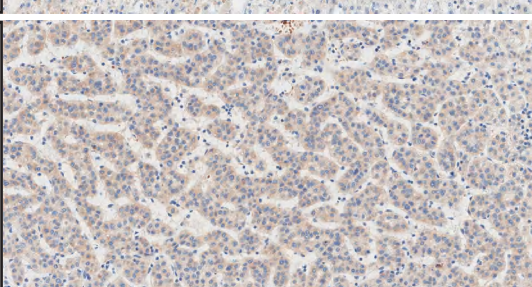 | 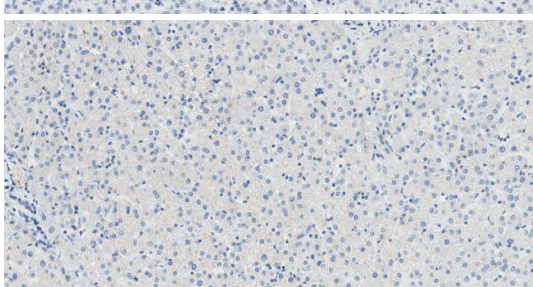 |

Supplement: Supplementary Figure 3 — The expression of EEF1E1 in HCC was detected by IHC(continued). [file DataSheet_3.pdf]

|           | Cancer                                                                              | Paracancerous                                                                        |
|-----------|-------------------------------------------------------------------------------------|--------------------------------------------------------------------------------------|
| Case VI   | 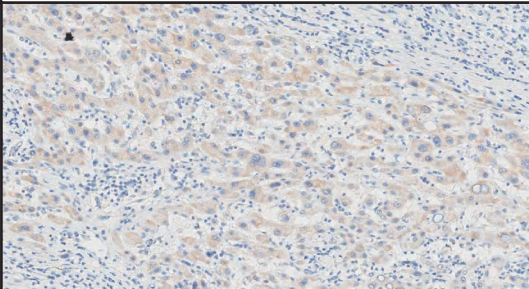    | 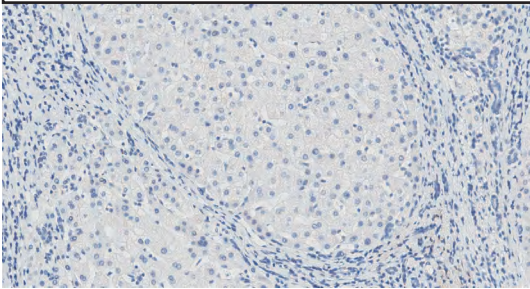    |
| Case VII  | 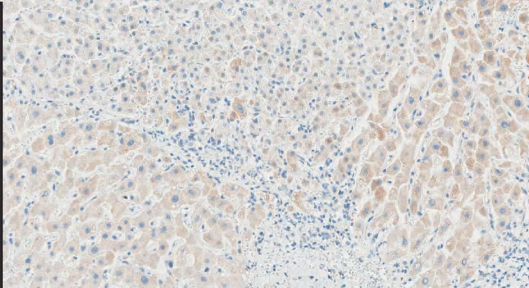   | 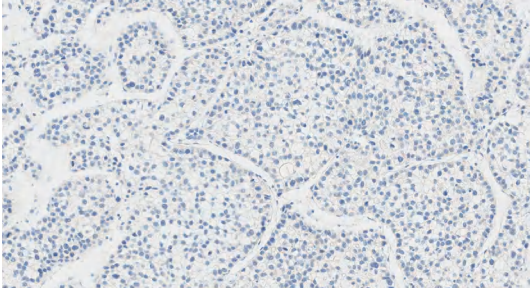   |
| Case VIII | 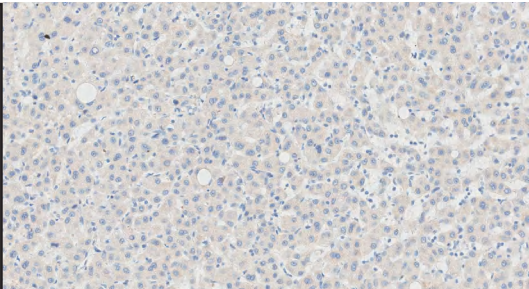   | 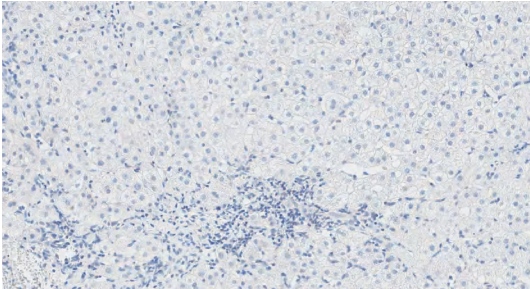   |
| Case IX   | 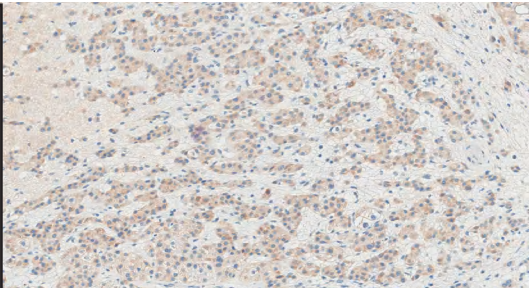  | 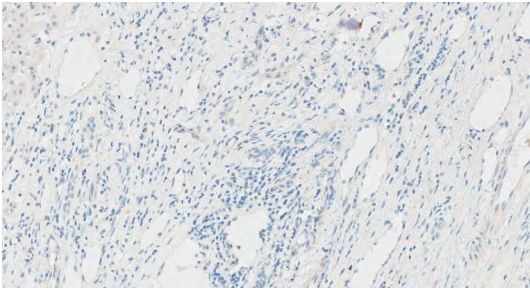  |
| Case X    | 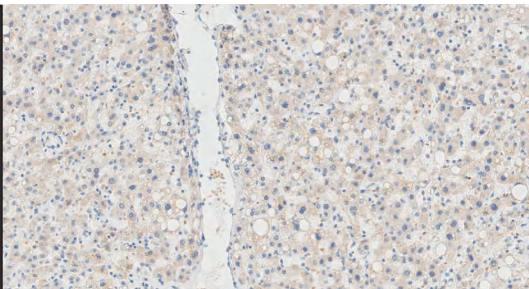 | 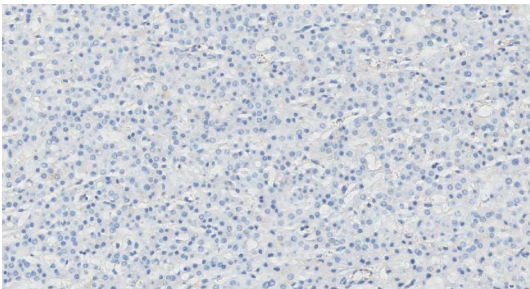 |

Supplement: Supplementary Figure 4 — The expression of EEF1E1 in HCC was detected by IHC(continued). [file DataSheet_4.pdf]

|           | Cancer                                                                              | Paracancerous                                                                        |
|-----------|-------------------------------------------------------------------------------------|--------------------------------------------------------------------------------------|
| Case XI   | 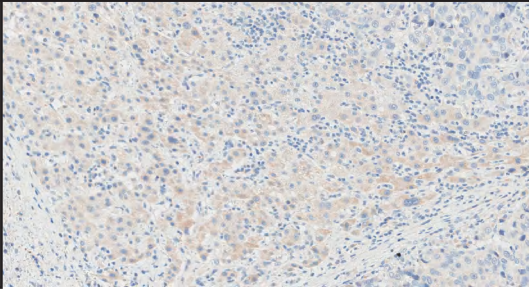    | 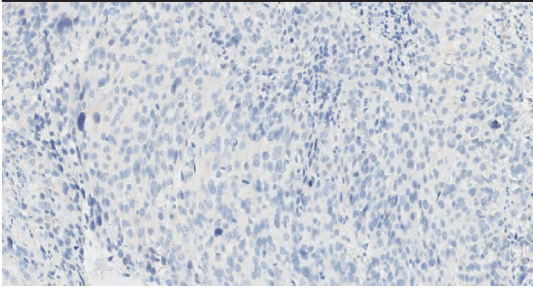    |
| Case XII  | 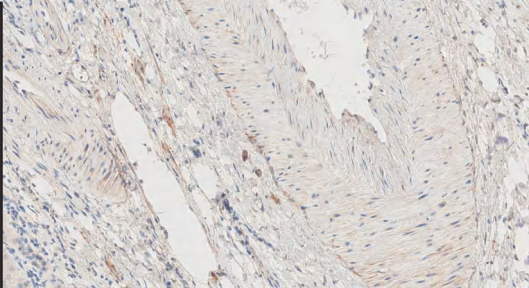   | 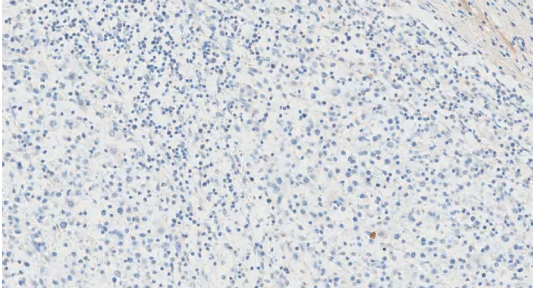   |
| Case XIII | 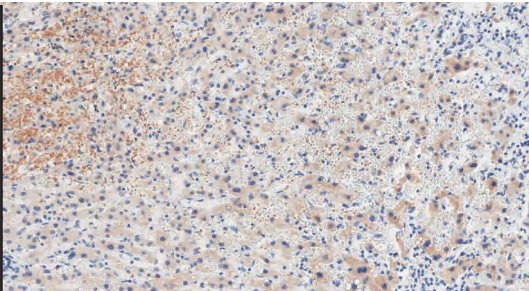   | 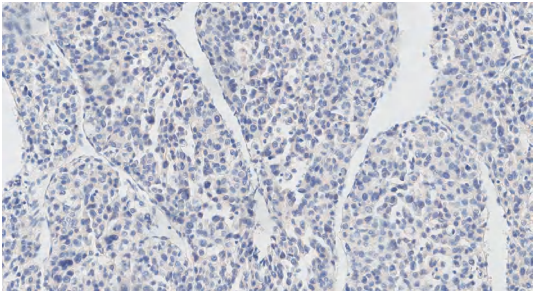   |
| Case XIV  | 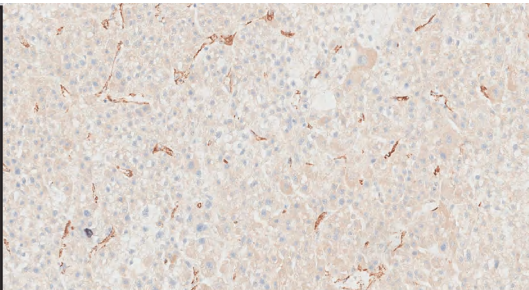  | 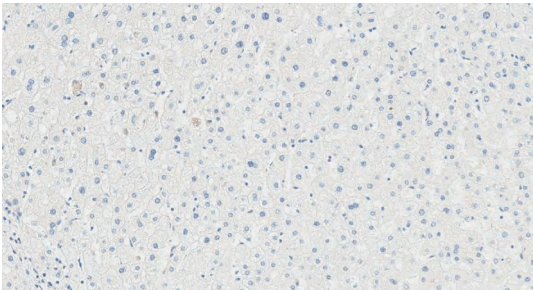  |
| Case XV   | 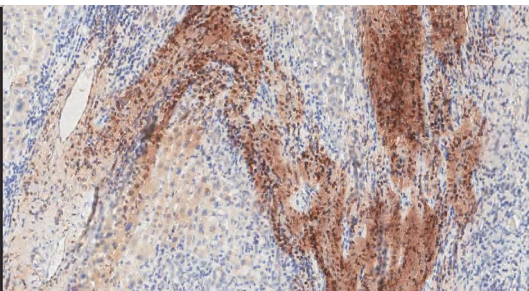 | 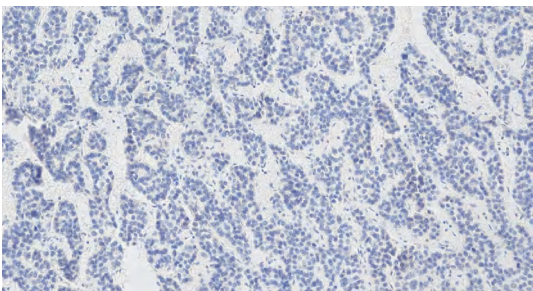 |

Supplement: Supplementary Figure 5 — The expression of EEF1E1 in HCC was detected by IHC(continued). [file DataSheet_5.pdf]

|            | Cancer                                                                              | Paracancerous                                                                        |
|------------|-------------------------------------------------------------------------------------|--------------------------------------------------------------------------------------|
| Case XVI   | 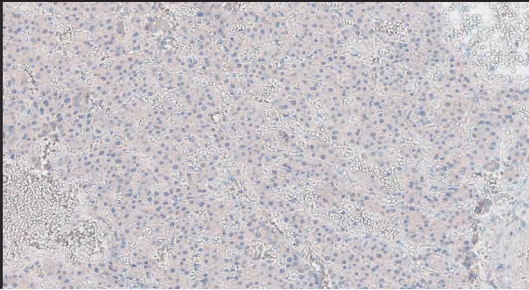    | 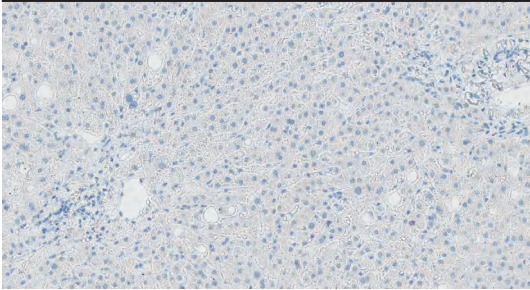    |
| Case XVII  | 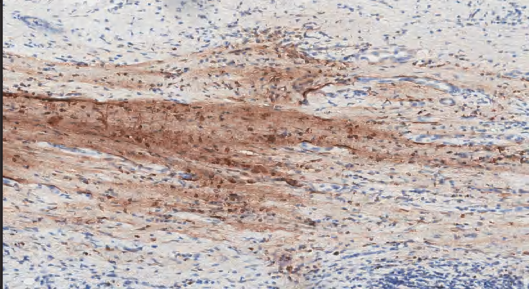   | 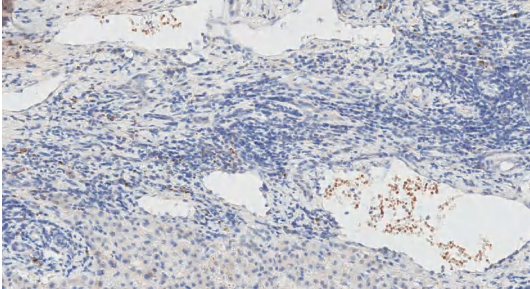   |
| Case XVIII | 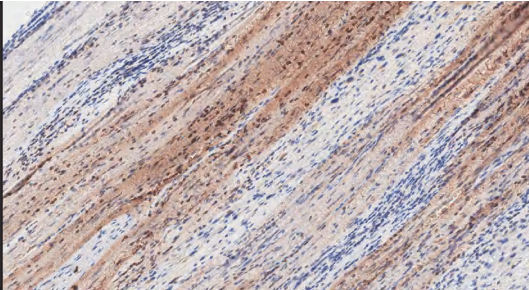   | 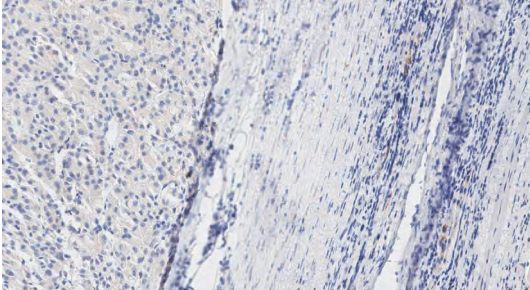   |
| Case XIX   | 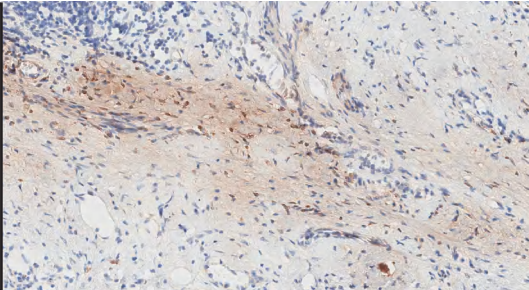  | 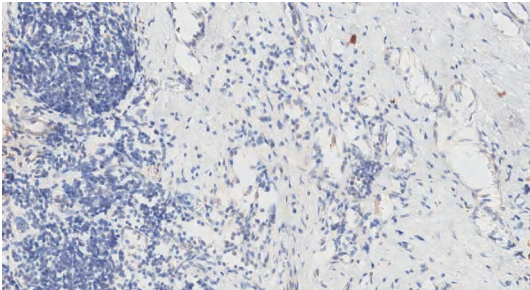  |
| Case XX    | 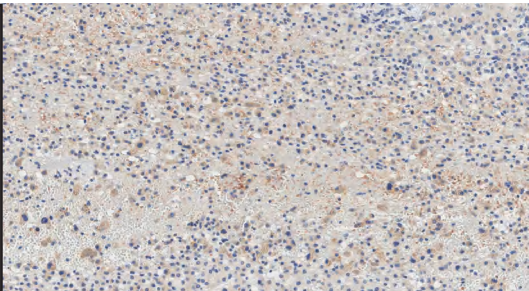 | 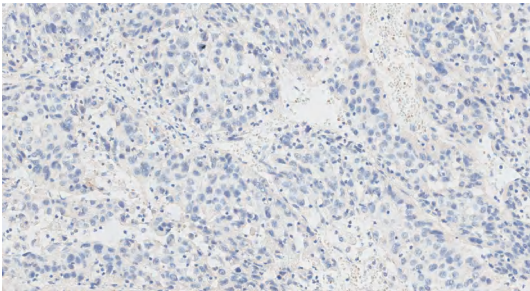 |

Supplement: Supplementary Figure 6 — The expression of EEF1E1 in HCC was detected by IHC(continued). [file DataSheet_6.pdf]

ATM expression in LIHC tissues

|         | Paracancerous                                                                       | Junction                                                                            | Cancer                                                                               |      |
|---------|-------------------------------------------------------------------------------------|-------------------------------------------------------------------------------------|--------------------------------------------------------------------------------------|------|
| case I  | 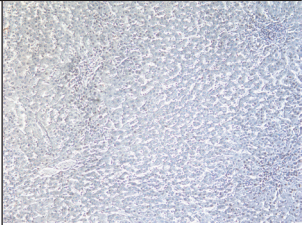   | 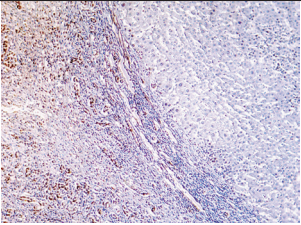   | 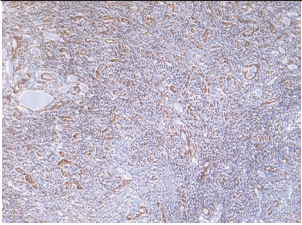   | 10 x |
|         | 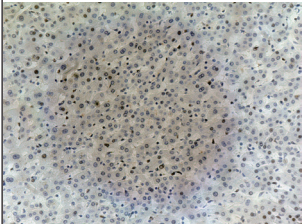   | 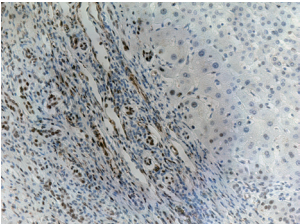   | 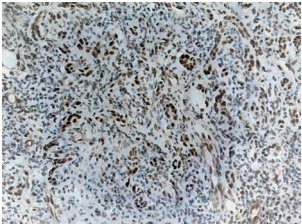   | 20 x |
|         | 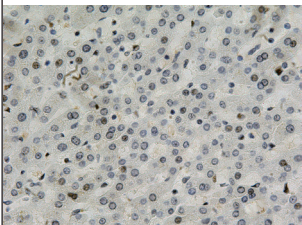   | 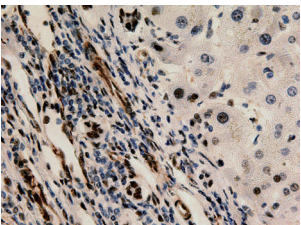   | 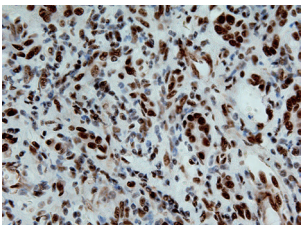   | 40 x |
| case II | 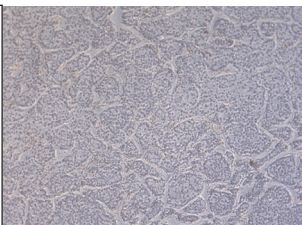  | 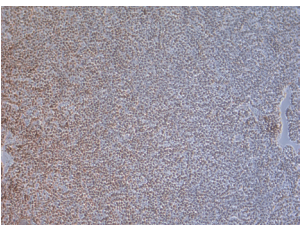  | 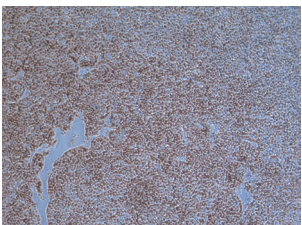  | 10 x |
|         | 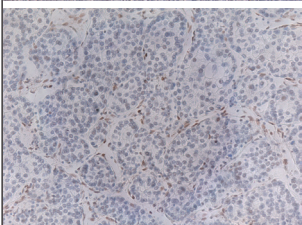 | 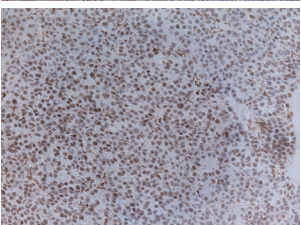 | 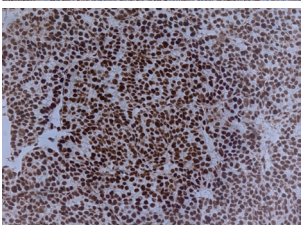 | 20 x |
|         | 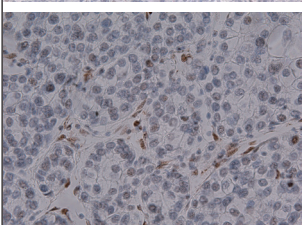 | 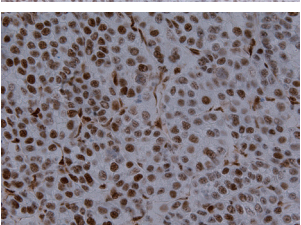 | 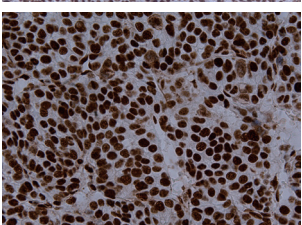 | 40 x |

Supplement: Supplementary Figure 7 — The expression of ATM in HCC was detected by IHC. [file DataSheet_7.pdf]

P53 expression in LIHC tissues

|         | Paracancerous                                                                       | Junction                                                                            | Cancer                                                                               |      |
|---------|-------------------------------------------------------------------------------------|-------------------------------------------------------------------------------------|--------------------------------------------------------------------------------------|------|
| case I  | 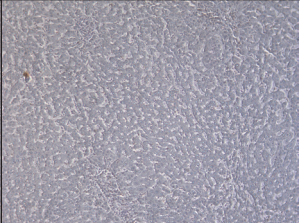   | 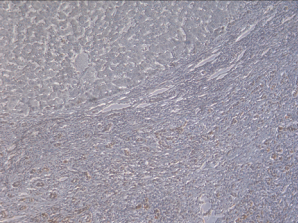   | 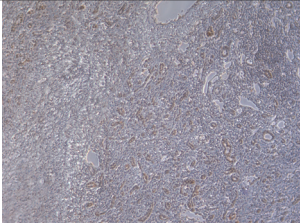   | 10 x |
|         | 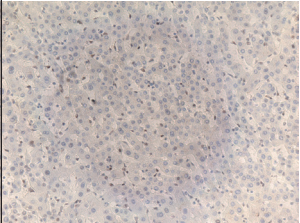   | 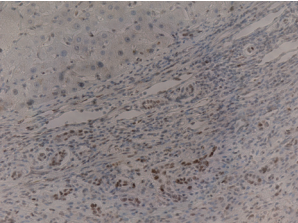   | 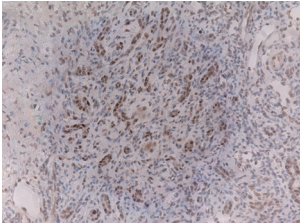   | 20 x |
|         | 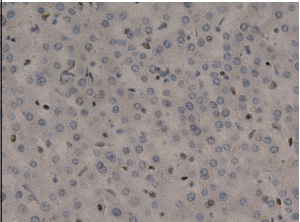   | 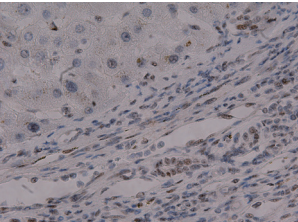   | 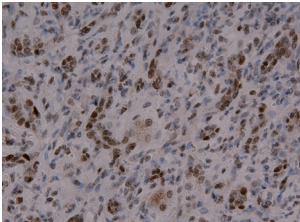   | 40 x |
| case II | 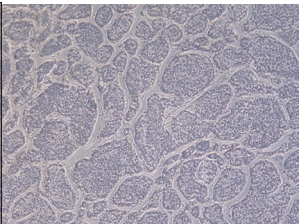  | 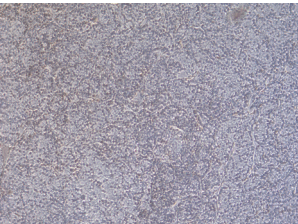  | 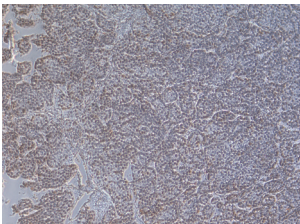  | 10 x |
|         | 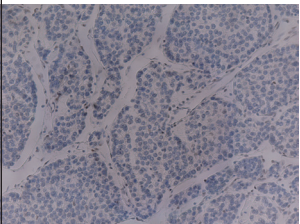 | 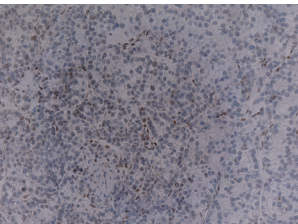 | 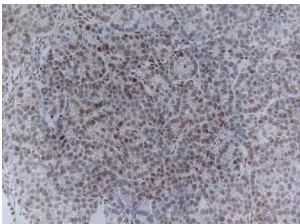 | 20 x |
|         | 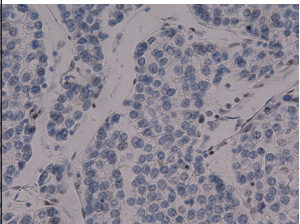 | 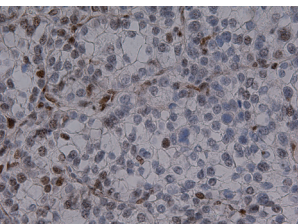 | 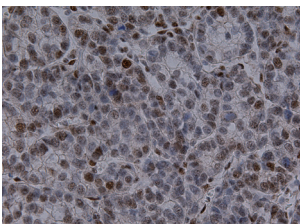 | 40 x |

Supplement: Supplementary Figure 8 — The expression of p53 in HCC was detected by IHC. [file DataSheet_8.pdf]

CASPASE3 expression in LIHC tissues

|         | Paracancerous                                                                       | Junction                                                                            | Cancer                                                                               |      |
|---------|-------------------------------------------------------------------------------------|-------------------------------------------------------------------------------------|--------------------------------------------------------------------------------------|------|
| case I  | 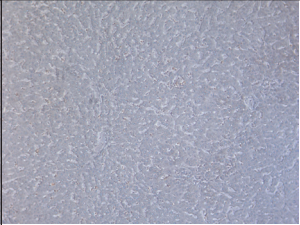   | 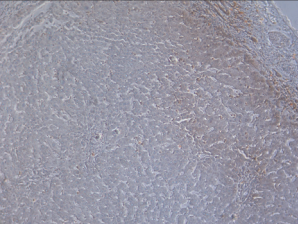   | 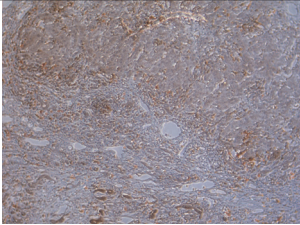   | 10 x |
|         | 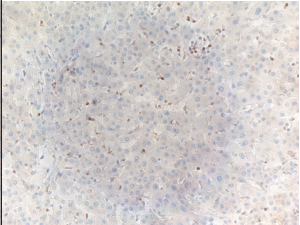   | 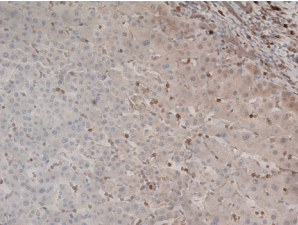   | 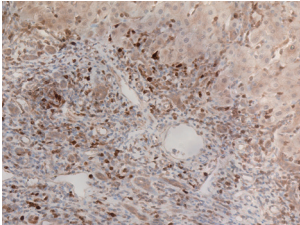   | 20 x |
|         | 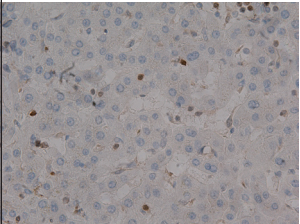   | 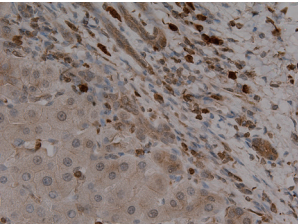   | 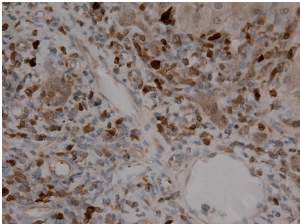   | 40 x |
| case II | 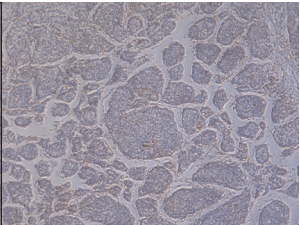  | 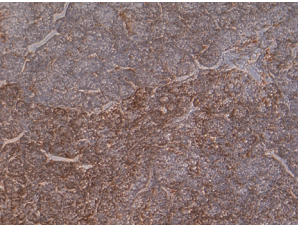  | 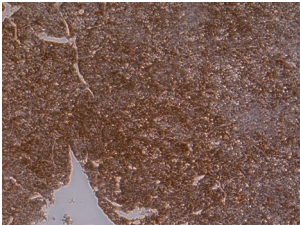  | 10 x |
|         | 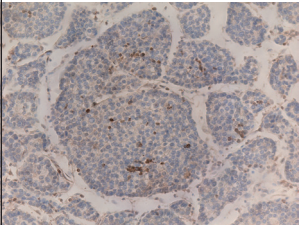 | 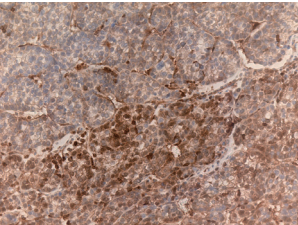 | 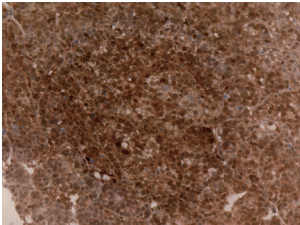 | 20 x |
|         | 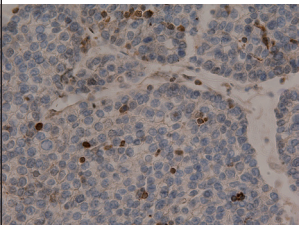 | 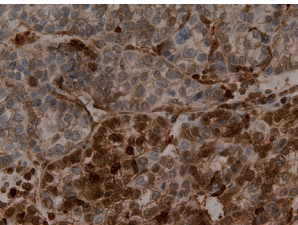 | 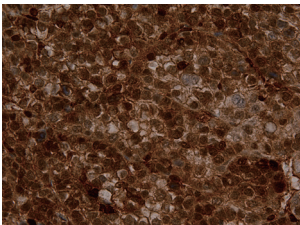 | 40 x |

Supplement: Supplementary Figure 9 — The expression of CASPASE3 in HCC was detected by IHC. [file DataSheet_9.pdf]

## CASE I    **EEF1E1 expression is associated with immune infiltration**

**EEF1E1**

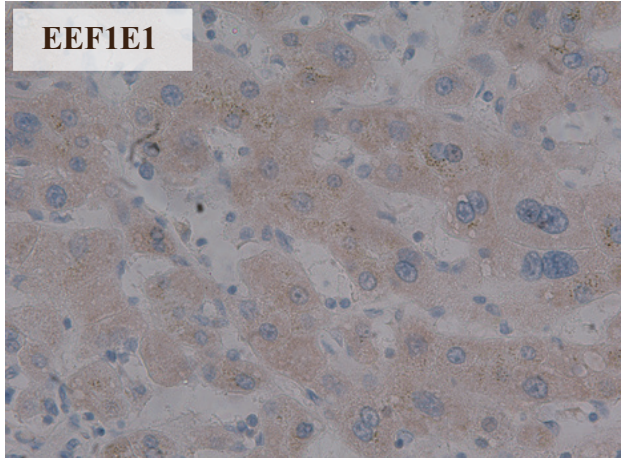

**CD3**

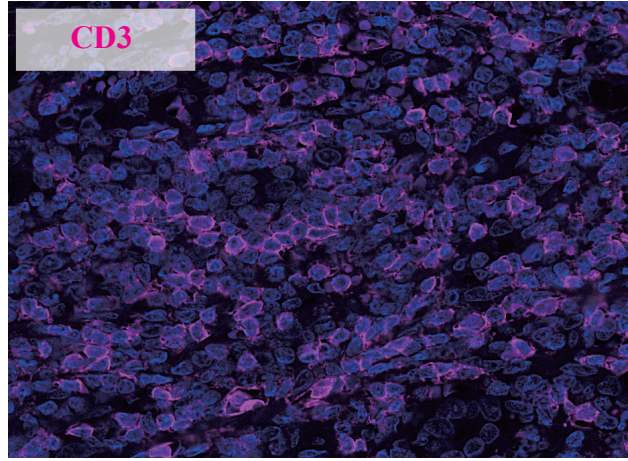

**CD4**

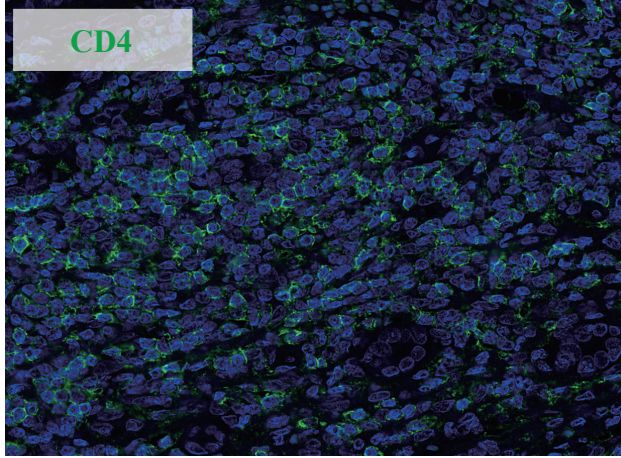

**CD8**

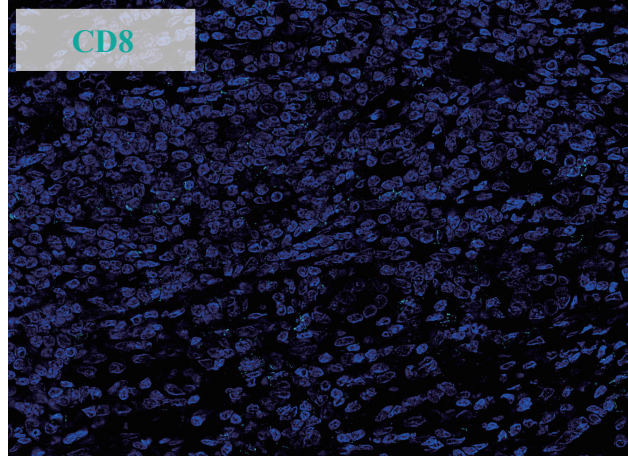

**PD1**

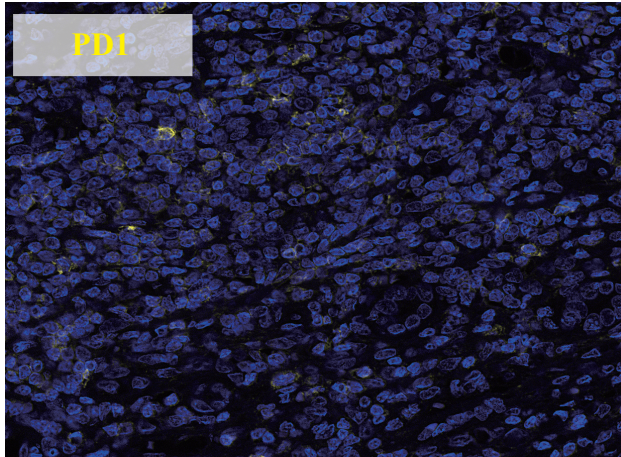

**MERGE**

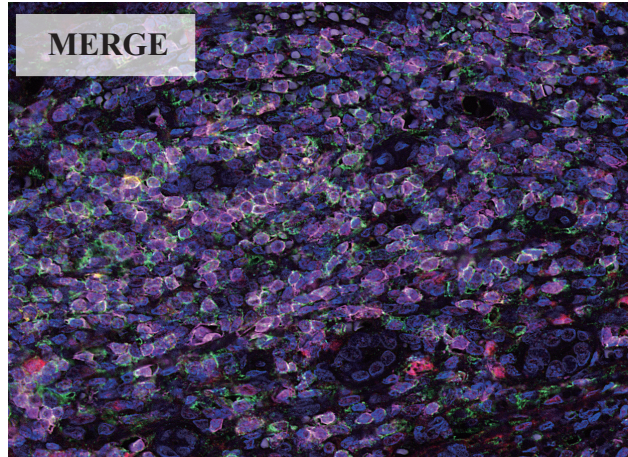

Supplement: Supplementary Figure 10 — Multiplex IHC detection the expression of CD3, CD4, CD8 and PD1 in HCC. [file DataSheet_10.pdf]

## CASE II EEF1E1 expression is associated with immune infiltration

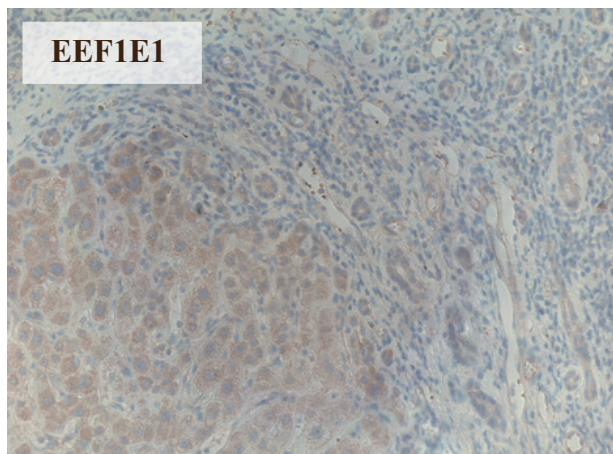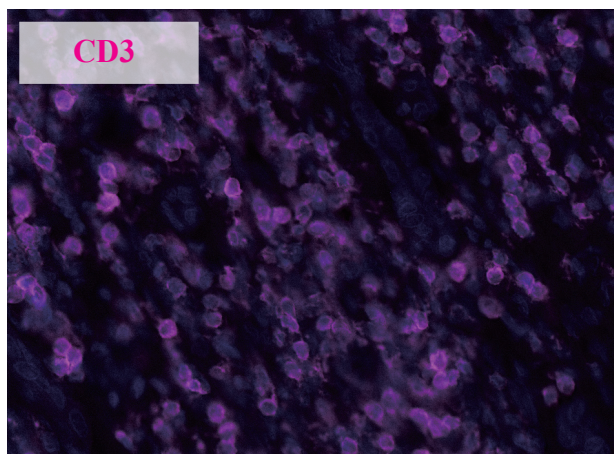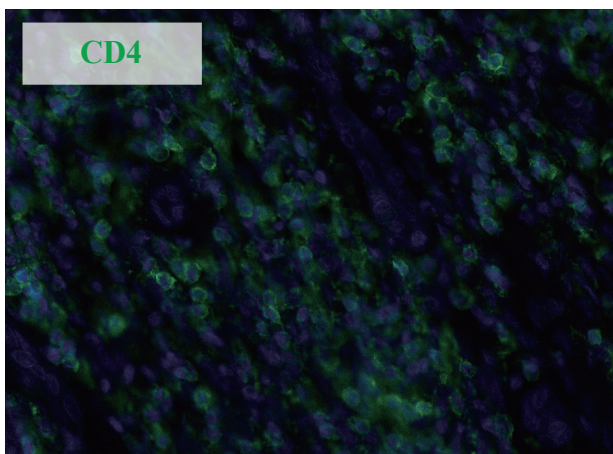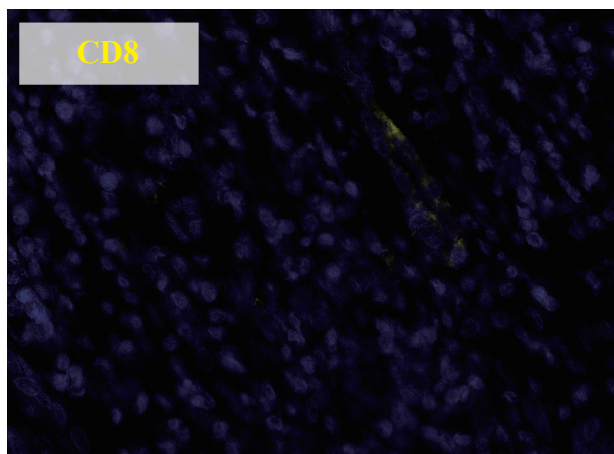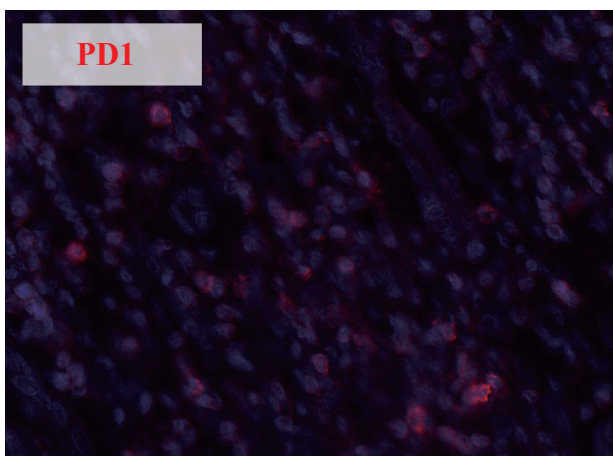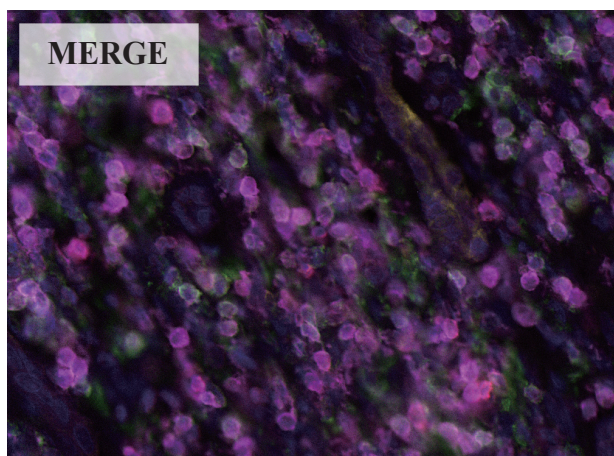

Supplement: Supplementary Figure 11 — Multiplex IHC detection the expression of CD3, CD4, CD8 and PD1 in HCC(continued). [file DataSheet_11.pdf]
